# Estimating intraseasonal intrinsic water‐use efficiency from high‐resolution tree‐ring δ^13^C data in boreal Scots pine forests

**DOI:** 10.1111/nph.18649

**Published:** 2022-12-19

**Authors:** Yu Tang, Elina Sahlstedt, Giles Young, Pauliina Schiestl‐Aalto, Matthias Saurer, Pasi Kolari, Tuula Jyske, Jaana Bäck, Katja T. Rinne‐Garmston

**Affiliations:** ^1^ Bioeconomy and Environment Unit Natural Resources Institute Finland (Luke) Latokartanonkaari 9 00790 Helsinki Finland; ^2^ Faculty of Agriculture and Forestry, Institute for Atmospheric and Earth System Research (INAR) / Forest Sciences University of Helsinki PO Box 27 00014 Helsinki Finland; ^3^ Faculty of Science, Institute for Atmospheric and Earth System Research (INAR) / Physics University of Helsinki PO Box 68 00014 Helsinki Finland; ^4^ Forest Dynamics Swiss Federal Institute for Forest, Snow and Landscape Research (WSL) Zürcherstrasse 111 8903 Birmensdorf Switzerland; ^5^ Production Systems Unit Natural Resources Institute Finland Tietotie 2 02150 Espoo Finland

**Keywords:** eddy covariance, intrinsic water‐use efficiency, laser ablation, leaf gas exchange, mesophyll conductance, *Pinus sylvestris* L., postphotosynthetic isotopic fractionation, tree‐ring δ^13^C

## Abstract

- Intrinsic water‐use efficiency (iWUE), a key index for carbon and water balance, has been widely estimated from tree‐ring δ^13^C at annual resolution, but rarely at high‐resolution intraseasonal scale.
- We estimated high‐resolution iWUE from laser‐ablation δ^13^C analysis of tree‐rings (iWUE_iso_) and compared it with iWUE derived from gas exchange (iWUE_gas_) and eddy covariance (iWUE_EC_) data for two *Pinus sylvestris* forests from 2002 to 2019.
- By carefully timing iWUE_iso_ via modeled tree‐ring growth, iWUE_iso_ aligned well with iWUE_gas_ and iWUE_EC_ at intraseasonal scale. However, year‐to‐year patterns of iWUE_gas_, iWUE_iso_, and iWUE_EC_ were different, possibly due to distinct environmental drivers on iWUE across leaf, tree, and ecosystem scales. We quantified the modification of iWUE_iso_ by postphotosynthetic δ^13^C enrichment from leaf sucrose to tree rings and by nonexplicit inclusion of mesophyll and photorespiration terms in photosynthetic discrimination model, which resulted in overestimation of iWUE_iso_ by up to 11% and 14%, respectively.
- We thus extended the application of tree‐ring δ^13^C for iWUE estimates to high‐resolution intraseasonal scale. The comparison of iWUE_gas_, iWUE_iso_, and iWUE_EC_ provides important insights into physiological acclimation of trees across leaf, tree, and ecosystem scales under climate change and improves the upscaling of ecological models.

## Introduction

How trees respond to climate change has profound impact on the carbon and water balances in forest ecosystems (Mathias & Thomas, 2021). This is because trees regulate stomata to control carbon dioxide uptake during photosynthesis and to control water loss that occurs with transpiration. The trade‐off between carbon gain and water loss can be quantified as intrinsic water‐use efficiency (iWUE), expressed as the ratio between net assimilation rate and stomatal conductance (Osmond *et al*., 1980). A common approach to estimate iWUE is via analysis of stable carbon isotope composition (δ^13^C) of annual tree rings (Farquhar *et al*., 1982, 1989). However, since tree‐ring δ^13^C reflects a time‐integrated signal (Cernusak, 2020), this method has been rarely applied for detecting high‐resolution intraseasonal variations in iWUE (but see Michelot *et al*., 2011).

Owing to recent advances in online methods for obtaining carbon isotope data from wood, via laser ablation coupled to an isotope ratio mass spectrometry (hereafter LA‐IRMS), the ease of determining the intraseasonal tree‐ring δ^13^C has been greatly improved (Soudant *et al*., 2016; Rinne‐Garmston *et al*., 2022). A pioneer study, which compared intraseasonal δ^13^C data of leaf sucrose and tree rings for *Larix gmelinii* (Rupr.), found similar low‐frequency trends for the two records but a systematic isotopic offset, caused by postphotosynthetic isotopic fractionation (Rinne *et al*., 2015). In future, it needs to be verified that such consistent, common patterns between sucrose and tree‐ring δ^13^C are also found for other species and site conditions. But considering that δ^13^C of leaf sucrose can accurately record iWUE at leaf level (Tang *et al*., in press) and that the sucrose δ^13^C signal is transported to and laid down in tree rings (Gessler *et al*., 2009), there is high potential to apply LA‐IRMS‐derived tree‐ring δ^13^C data for estimating iWUE at intraseasonal scale. As δ^13^C signal can be potentially retrieved from tree‐ring archives which cover a wide range of areas and periods, such applications, if successful, will be of high value to study the short‐term dynamics of CO_2_ and H_2_O trade, especially for areas and periods without instrumental records.

For a reliable and accurate estimation of iWUE from tree‐ring δ^13^C (hereafter iWUE_iso_), it is important to quantify how a δ^13^C signal may be altered from leaf assimilates to tree rings in postphotosynthetic processes (Fiorella *et al*., 2022). These processes include the use of reserves in early growing season (McCarroll *et al*., 2017; Fonti *et al*., 2018), isotopic fractionation associated with metabolic processes (Gessler *et al*., 2009; Rinne *et al*., 2015), and integration of phloem sugars that are assimilated at different canopy heights (Schleser, 1990; Bögelein *et al*., 2019). These processes are suggested to be species‐specific and site‐specific. For example, the use of reserves has been detected for *Quercus petraea* at a temperate forest in Fontainebleau‐Barbeau (Vincent‐Barbaroux *et al*., 2019), but not for *Larix gmelinii* Rupr. in the permafrost zone of Central Siberia (Rinne *et al*., 2015). Furthermore, whereas phloem sugars at breast height originated largely from the upper crown for *Fagus sylvatica*, they originated mainly from the inner and self‐shaded crown parts for *Pseudotsuga menziesii* (Bögelein *et al*., 2019). For a certain site or tree species, concomitant high temporal tracking of leaf sucrose δ^13^C along with tree‐ring δ^13^C measurements can help to quantify the extent of postphotosynthetic δ^13^C modification (*f*_post_) and the consequent impact on the iWUE_iso_ estimates. Such information can also be used as a guideline in studies conducted under similar growth conditions for the same species.

The accuracy of iWUE_iso_ calculation may also be improved by the use of a complex photosynthetic discrimination model, for instance, via the explicit consideration of mesophyll (Gimeno *et al*., 2021; Ma *et al*., 2021) and photorespiratory effects (Keeling *et al*., 2017; Schubert & Jahren, 2018). Nevertheless, implementing the complex version of photosynthetic discrimination model can be difficult (Lavergne *et al*., 2019, 2022), partly due to a limited understanding of mesophyll conductance (*g*_m_) and photorespiration dynamics, both of which are dependent on plant species and leaf environment (Sun *et al*., 2014; Schubert & Jahren, 2018). Hence, the simplified model (Farquhar *et al*., 1982, 1989) has been applied in the majority of iWUE_iso_ reconstruction studies (e.g. Frank *et al*., 2015; Guerrieri *et al*., 2019). However, it is worthwhile to evaluate how mesophyll and photorespiratory effects may impact iWUE_iso_ estimates, as this may help to reconcile the trends and absolute values of iWUE derived from different methods.

Intrinsic water‐use efficiency can be also estimated from gas exchange and eddy covariance (EC) measurements (iWUE_gas_ and iWUE_EC_, respectively; e.g. Keenan *et al*., 2013; Medlyn *et al*., 2017). iWUE_gas_, iWUE_iso_, and iWUE_EC_ represent signals at different scales: leaf level, whole‐tree level, and ecosystem level, respectively. Comparisons between these ‘scale‐specific’ methods not only reveal the limitations of each method (Medlyn *et al*., 2017) but also show promise to cross‐validate different sources of iWUE data (Guerrieri *et al*., 2019). Since the gas exchange and EC data are of high temporal resolution, they can overall help verify the intraseasonal pattern of iWUE derived from tree‐ring δ^13^C data, albeit uncertainties exist in each iWUE estimation method (Medlyn *et al*., 2017; Knauer *et al*., 2018; Lavergne *et al*., 2019). The three methods have been scantly compared for their absolute iWUE values, at a global scale for different plant functional types (Medlyn *et al*., 2017) or at a local site for different tree species (Yi *et al*., 2019). Studies that compare temporal changes in these iWUE estimates have not been published at intraseasonal scale, and few exist at interannual resolution (Guerrieri *et al*., 2019; Lavergne *et al*., 2019). Previous studies have demonstrated a site‐specific (Martínez‐Sancho *et al*., 2018; Marchand *et al*., 2020) and temporally dynamic (Liu *et al*., 2014; Wieser *et al*., 2018) response of interannual iWUE to environmental drivers. Further knowledge on the scale‐specific temporal trends of iWUE is not only valuable for in‐depth understanding of tree physiological responses to environmental change but also can improve the upscaling of ecological models.

The main objective of this study was to evaluate the reliability of high‐resolution tree‐ring δ^13^C data to estimate intraseasonal changes in iWUE. For this purpose, we (1) compared the intraseasonal trends and absolute values of iWUE_iso_, derived from tree‐ring LA‐IRMS δ^13^C analysis, with that of iWUE_EC_ and iWUE_gas_. The comparison was made using a unique set of EC and gas exchange data covering the period from 2002 to 2019, at two Scots pine‐dominated boreal forests with contrasting growth conditions. Next, in the effort to reconcile differences between absolute values of the three iWUE series, we (2) quantified the impact of *f*_post_, *g*_m_, and photorespiration on iWUE_iso_ estimates. Furthermore, we (3) discussed the environmental and physiological controls on iWUE_iso_, iWUE_gas_, and iWUE_EC_ at both intraseasonal and interannual scales, and strengths and weaknesses of each iWUE estimation method.

## Materials and Methods

### Site description and environmental data

The study was conducted at two boreal forests dominated by Scots pine (*Pinus sylvestris* L.) in northern and southern Finland (Fig. S1), both of which belong to the Stations for Measuring Ecosystem‐Atmosphere Relations (SMEAR) network. The northern site, Värriö, is close to the arctic‐alpine timberline for Scots pine. The growth conditions are harsher in Värriö, evident in the lower tree heights, sparser canopy (Fig. S1), lower temperature, and shorter growing season, compared with Hyytiälä (Table S1). More characteristics of the study sites are listed in Table S1.

Air temperature (*T*) and relative humidity (RH) at the canopy height (16.8 m in Hyytiälä and 9 m in Värriö), precipitation, soil moisture at the topsoil, and air pressure (*P*_a_) at ground level were retrieved from the AVAA Smart SMEAR portal (https://smear.avaa.csc.fi/). Vapor pressure deficit (VPD) was calculated from *T* and RH. Cumulative precipitation and the means of other environmental variables at half‐hourly scale and during the daytime, which was defined as the period from 2 h after sunrise to 2 h before sunset, were calculated.

### Sampling and δ^13^C analysis

#### Sampling

To determine *f*_post_ from leaf sugars to phloem sugars and eventually to tree rings, we collected needle and phloem samples during the season 2018 at both sites. One‐year‐old needles (1 N) and current‐year needles (0 N) were collected every 1 or 2 wk from the sun‐exposed top canopy of five mature trees for sugar δ^13^C analysis. Sampling started before the onset of radial growth (early May in Hyytiälä and late May in Värriö) and ended after the cessation of radial growth (October in both sites), conducted all together 20 times per site. Phloem samples were collected at breast height from five mature trees on 6 d per site and season, twice in May and once per month from June to September. Needle and phloem samples were put in a cool box immediately upon collection, and microwaved at 600 W for 1 min within 2 h to stop metabolic activities (Wanek *et al*., 2001). Tree‐ring samples were taken at breast height after the cessation of growth in 2019. In Värriö, one 5‐mm‐diameter core sample was collected from five mature trees, while in Hyytiälä, five trees were felled, and cross sections were obtained. Average δ^13^C from five trees was used to represent the average conditions experienced by trees at the study sites (Leavitt & Long, 1984). All sampled trees were within the 80% footprint boundaries of the EC towers, which are *c*. 400 m in Hyytiälä (Launiainen *et al*., 2022) and 200 m in Värriö (Kulmala *et al*., 2019).

#### δ^13^C of leaf and phloem sugars

Water‐soluble carbohydrates (WSCs) were extracted and purified from homogenized needle and phloem samples, according to Wanek *et al*. (2001) and Rinne *et al*. (2012). Briefly, the supernatant was separated from the water extraction at 85°C and then purified by three types of sample preparation cartridges (Dionex OnGuard II H, A and P; Thermo Fisher Scientific, Waltham, MA, USA). The purified WSCs were lyophilized, dissolved in deionized water, and filtered through a 0.45‐μm syringe filter (Acrodisc).

δ^13^C of WSCs was measured using an elemental analyzer (EA; Europa EA‐GSL; Sercon Ltd, Crewe, UK) coupled to an IRMS (20–22 IRMS; Sercon Ltd) at the Stable Isotope Laboratory of Luke (SILL) at Natural Resources Institute Finland (Luke, Helsinki). Before δ^13^C analysis, aliquots of solubilized WSCs were pipetted into individual tin capsules (IVA Analysentechnik, Meerbusch, Germany), freeze‐dried, and wrapped. Three reference materials were used to calibrate the δ^13^C values of WSCs, IAEA‐CH3 (cellulose, −24.724‰), IAEA‐CH7 (polyethylene, −32.151‰), and an in‐house sucrose reference (Sigma‐Aldrich, −12.22‰). Repeat measurement of a quality control material indicates a measurement precision of 0.1‰ (SD). δ^13^C of leaf WSCs was calculated as the average δ^13^C of WSCs in 1 N and 0 N.

δ^13^C values of sucrose were analyzed at WSL (Birmensdorf, Switzerland), using a Delta V Advantage IRMS (Thermo Fisher Scientific) coupled with a high‐performance liquid chromatography (HPLC) system with a Finnigan LC Isolink interface (Thermo Fisher Scientific) (Rinne *et al*., 2012). External sucrose standards, with known δ^13^C values and comparable concentrations to samples (from 20 to 180 ng C μl^−1^), were analyzed every 10 samples. Correction for HPLC‐IRMS δ^13^C values was performed according to Rinne *et al*. (2012). The measurement precision of sucrose standards was 0.26‰ (SD). δ^13^C of leaf sucrose was calculated as the average δ^13^C of sucrose in 1 N and 0 N.

#### δ^13^C of tree rings

Tree‐ring samples were air‐dried before the preparation for isotope analysis. Each sample was sanded with progressively finer grades of sandpaper until ring boundaries and individual cells were clearly identifiable. To ensure that any sawdust collected in voids and intercellular spaces did not affect the isotope signal, each sample was placed in distilled water in an ultrasonic bath for 30 min to remove the sawdust. Samples were then visually inspected under a binocular microscope. Tree rings were measured using WinDendro™ and statistically crossmatched against local chronologies to ensure the correct year was assigned to each ring. Mobile resin and extractives of the samples were removed using a 2 : 1 mixture of toluene and ethanol in a Soxhlet extractor for a duration of 48 h (Loader *et al*., 1997). After extraction, any residual toluene and ethanol in the samples were removed by rinsing the samples with distilled water in the Soxhlet extractor. Samples were then air‐dried. Resin‐extracted wood was used for LA‐IRMS analysis, as suggested by Schulze *et al*. (2004).

**Fig. 1:**
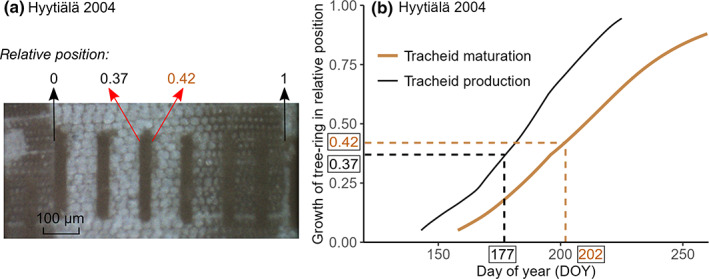
Example of how the formation period of a tree‐ring δ^13^C measurement of Scots pine was defined. (a) Relative position of a tree‐ring δ^13^C measurement within a tree ring; (b) formation period of the tree‐ring δ^13^C measurement based on the growth curves of tracheid production and tracheid maturation for the specific year and site. The growth curves were modeled via the Carbon Allocation Sink Source Interaction model (Schiestl‐Aalto *et al*., 2015). First, the relative position of a δ^13^C measurement within a tree ring was defined, for example from 0.37 to 0.42 in (a). Then, the initial development date of the tracheids representing that δ^13^C measurement was determined, according to the tracheid production curve in (b) (day of year, DOY) 177 for relative position 0.37). Next, the date was defined when the tracheids for that δ^13^C measurement were fully mature, according to the tracheid maturation curve (DOY 202 for relative position 0.42). Finally, the obtained period representing a tree‐ring δ^13^C value and intrinsic water‐use efficiency derived therefrom (iWUE_iso_ ), in this case from DOY 177 to DOY 202, was used to align iWUE_iso_ with iWUE derived from eddy covariance (iWUE_EC_ ) and gas exchange (iWUE_gas_ ) data.

Intraseasonal δ^13^C was analyzed for each tree‐ring sample using LA‐IRMS at SILL (Methods S1), following the operation principle by Schulze *et al*. (2004) and Loader *et al*. (2017). In brief, ablated dust particles were carried by helium flow through a combustion device, the resulting CO_2_ was collected with liquid nitrogen, and subsequently, the CO_2_ was released upon heating and purified in a GC‐column before its introduction to IRMS. A series of 40 μm tangential laser tracks were sampled along the same radial direction on tree rings at an interval of 40 μm (years from 2010 to 2019) or 80 μm (Fig. 1a). Depending on the ring width, between 5 and 33 laser tracks per tree ring were ablated for δ^13^C analysis. Each sample was run against an in‐house CO_2_ reference gas. The raw δ^13^C values were calibrated against USGS‐55 (Mexican ziricote tree powder, −27.13‰) and an in‐house reference (yucca plant powder, −15.46‰), which were both measured concurrently with the tree‐ring samples. The USGS‐55 and the in‐house reference were in the form of a 10‐mm‐diameter disk, which had been prepared by compressing powder using a manual hydraulic press, providing a smooth, solid surface for ablation. In addition, IAEA‐C3 cellulose paper was measured multiple times during each run for quality control of the produced δ^13^C values. The LA‐IRMS measured δ^13^C value for IAEA‐C3 was −24.69 ± 0.24‰, which is in line with the certified value of −24.91 ± 0.49‰. Spot sizes and track lengths of the reference materials were varied to produce variation in signal size, which enabled monitoring of a size effect on δ^13^C values, and the data were corrected when needed (Werner & Brand, 2001).

### Averaging and timing intraseasonal δ^13^C

Individual tree‐ring δ^13^C series had in general similar intraseasonal patterns (Figs S2, S3), enabling the calculation of site‐representative tree‐ring δ^13^C series. First, considering tree‐to‐tree differences in tree‐ring widths, we aligned the intraseasonal tree‐ring δ^13^C data against their relative position within a tree ring (from 0 to 1) per tree, year, and site. Then, we interpolated tree‐ring δ^13^C per tree, year, and site against relative position from 0.05 to 1 at an interval of 0.05 (Figs S2, S3). Finally, we calculated site‐representative tree‐ring δ^13^C as averages of five tree rings at the interpolated relative position.

To time intraseasonal tree‐ring δ^13^C, tree‐ring growth curves against day of year (DOY) were simulated per year and site via a dynamic growth model *Carbon Allocation Sink Source Interaction* (CASSIA; Schiestl‐Aalto *et al*., 2015). The performance of CASSIA model results was evaluated by comparison with xylogenesis observational results for years 2007, 2008, 2009, 2018, and 2019, for both sites (Methods S2; Fig. S4). With simulated growth curves, the start DOY of tracheid production and the end DOY of tracheid maturation could be determined for each site‐representative tree‐ring δ^13^C data point (Fig. 1).

### iWUE estimates

#### iWUE from leaf gas exchange (iWUE_gas_ )

CO_2_ fluxes (*A*) and H_2_O fluxes (*E*) were measured online with leaf gas exchange systems, as described by Altimir *et al*. (2002) and Aalto *et al*. (2014) from 2002 to 2019 at both sites. However, no data were available for years 2005 and 2014 in Hyytiälä. In brief, transparent acrylic plastic chambers were installed at the top canopy of one to four mature trees with a debudded 1‐ or 2‐yr‐old shoot enclosed. Four chamber designs were employed over the years in Hyytiälä, whereas the chamber design in Värriö was the same all the time. The nonairtight chambers were automatically closed intermittently for 50 to 80 times (in Hyytiälä) or 150 to 180 times (in Värriö) per day, with sample air drawn to gas analyzers (URAS‐4; Hartmann & Braun, Siek, Germany; LI‐840; Li‐Cor, Lincoln, NE, USA). CO_2_ fluxes and *E* were calculated from instantaneous CO_2_ and H_2_O records taken during the first 30–40 s of chamber closure (Kolari *et al*., 2012). Flux data were omitted when RH exceeded 85% to avoid biased results due to adsorption of water on chamber walls and tubing (Altimir *et al*., 2006). Small fluxes (*A* < 0.5 μmol m^−2^ s^−1^ and *E* < 0.1 mmol m^−2^ s^−1^) were also discarded given the uncertainties they may cause in the calculation of iWUE_gas_. Half‐hourly *A* and *E* data series were produced and applied to calculate iWUE_gas_ (Eqn 1; Beer *et al*., 2009), where *g* is stomatal conductance. In the well‐stirred chambers, boundary layer conductance is high (Uddling & Wallin, 2012) and thus for simplicity not considered in the calculation of iWUE_gas_.

(Eqn 1) $${\text{iWUE}}_{\mathrm{gas}}=A/g=A/E\cdot \left(\mathrm{VPD}/P_{a}\right)$$

#### iWUE from eddy covariance (iWUE_EC_ )

The net ecosystem CO_2_ exchange (NEE) and H_2_O flux (ET) were measured using a closed‐path EC system above the stand at 24 m height from 2002 to 2017 and at 27 m height from 2018 to 2019 in Hyytiälä, and at 16.6 m height from 2012 to 2019 in Värriö. Briefly, the EC data were: screened for outliers and erroneous measurements using standard methods (Aubinet *et al*., 2012); filtered by the turbulence criteria (Markkanen *et al*., 2001); averaged to half‐hourly scale and gap‐filled (Kulmala *et al*., 2019); and corrected for the storage of CO_2_ below the measuring height (Kolari *et al*., 2009; Launiainen *et al*., 2016). Half‐hourly gross primary production (GPP) was calculated by subtracting the modeled total ecosystem respiration from NEE (Kulmala *et al*., 2019). Furthermore, half‐hourly GPP and ET data were discarded, when precipitation occurred before the measurements, or when RH was higher than 85% to minimize the effect of condensation on canopy surfaces or instruments. More detailed description of the EC systems can be found in Vesala *et al*. (2005) and Kulmala *et al*. (2019), and EC data processing in Launiainen *et al*. (2016) and Mammarella *et al*. (2016). Assuming infinite aerodynamic conductance and no contribution of nontranspiratory water fluxes, half‐hourly iWUE_EC_ was calculated from GPP and ET data by Eqn 2 (Beer *et al*., 2009), where *G*_s_ is surface conductance. Twenty‐three percentage of half‐hourly EC data were gap‐filled. Using the gap‐filled data had limited impact on the temporal trends and absolute values of iWUE_EC_, considering that the iWUE_EC_ series with and without days that had high percentage (≥ 50%) of gap‐filled flux records were highly correlated (*r* = 0.98, *P* < 0.001).

(Eqn 2) $${\text{iWUE}}_{\mathrm{EC}}=\mathrm{GPP}/G_{s}=\mathrm{GPP}/\mathrm{ET}\cdot \left(\mathrm{VPD}/P_{a}\right)$$

#### iWUE from tree‐ring δ^13^C (iWUE_iso_ )

Intrinsic water‐use efficiency can also be estimated from isotope data via the photosynthetic discrimination model of Farquhar *et al*. (1982, 1989) (Eqns (Eqn 3), (Eqn 4), (Eqn 5), (Eqn 6)).

(Eqn 3) $${\text{iWUE}}_{\mathrm{iso}}=\left(c_{a}-c_{i}\right)/1.6$$

(Eqn 4) $$\Delta =a+\left(b-a\right)\cdot \frac{c_{i}}{c_{a}}-\left(b-a_{m}\right)\cdot A/\left(g_{m}\cdot c_{a}\right)-f\cdot \Gamma_{*}/c_{a}$$

(Eqn 5) $$\Gamma_{*}=42.75\cdot \exp\left[37830\cdot \left(T_{k}-298\right)/\left(298\cdot R\cdot T_{k}\right)\right]$$

(Eqn 6) $$\Delta =\left(\delta^{13}C_{\mathrm{air}}-\delta^{13}C_{\text{tree}}\right)/\left(1+\delta^{13}C_{\text{tree}}/1000\right)$$

*c*_a_ and *c*_i_ are the atmospheric and intercellular CO_2_ concentrations, respectively; Δ is the photosynthetic discrimination; *a* (4.4‰) is the fractionation due to diffusion of CO_2_ through stomata; *b* (29‰) is the fractionation due to carboxylation; *a*_m_ (1.8‰) is the fractionation during the mesophyll CO_2_ transfer; *f* is the fractionation during photorespiration; $\Gamma_{*}$ is the CO_2_ compensation point in the absence of dark respiration in μmol mol^−1^, estimated according to Eqn 5 (Bernacchi *et al*., 2001); *T*_k_ is the leaf temperature in K, taken as the air *T* measured inside the chamber; R is the universal gas constant (8.3145 J mol^−1^ K^−1^); δ^13^C_air_ is the δ^13^C of atmospheric CO_2_; and δ^13^C_tree_ is the site‐representative δ^13^C of the tree rings (resin‐extracted whole wood). Event‐based *c*_a_ and δ^13^C_air_ values in a northern Finnish site, Pallas (67°58′N, 24°7′E, 565 m asl (above sea level), https://gml.noaa.gov/dv/site/PAL.html), the closest site with continuous records for both *c*_a_ and δ^13^C_air_, were interpolated to daily scale and used. As there were no δ^13^C_air_ data observed during 2015 to 2019 in Pallas, we estimated δ^13^C_air_ from *c*_a_ and the linear regression between δ^13^C_air_ and *c*_a_ in Pallas (Fig. S5). The average *c*_a_ and δ^13^C_air_ for the formation period of each tree‐ring δ^13^C measurement were used as the input for Eqns (Eqn 3), (Eqn 4), (Eqn 6). Fractionation associated with day respiration was not considered here, considering that day respiration is intensively inhibited (Keenan *et al*., 2019) and has insignificant impact on Δ when net assimilation rate is high (Busch *et al*., 2020).

To test the impact of mesophyll and photorespiratory terms on iWUE_iso_, we took following assumptions: with no explicit consideration of *g*_m_ and *f*, by setting *g*_m_ = ∞, *f* = 0, and *b =* 27‰, which is the simplified model widely used for estimating iWUE_iso_; constant *g*_m_ of 0.127 mol m^−2^ s^−1^, corrected for all‐sided leaf area (Stangl *et al*., 2019) and *f* = 8‰ (Ghashghaie *et al*., 2003); constant *g*_m_ and *f* = 16‰ (Evans & von Caemmerer, 2013); dynamic *g*_m_ varying with *T* (Methods S3; Sun *et al*., 2014) and *f* = 8‰; dynamic *g*_m_ and *f* = 16‰. We compared how the absolute values of iWUE_iso_ changed across different assumptions, and evaluated whether the intraseasonal variations or interannual trends of iWUE_iso_ varied between different assumptions. In later sections, iWUE_iso_ from the simplified model was reported, unless otherwise specified.

### Data analysis

To align iWUE_gas_, iWUE_EC_, and iWUE_iso_ at intraseasonal scale, we averaged daytime iWUE_gas_ and iWUE_EC_ over the period representing each iWUE_iso_ estimate (Fig. 1). For simplicity, we did not consider changes in carbon allocation rate over time. Phloem transport time from top canopy to breast height was set to 2 d in Hyytiälä and 1 d in Värriö, according to Mencuccini & Hölttä (2010), that is, each period representing an iWUE_iso_ estimate (Fig. 1) was corrected for these lags. Due to differences in absolute values of iWUE and a more dampened trend in iWUE_iso_ in comparison with the other records, iWUE series were *z*‐scored per site, year, and method for intraseasonal comparisons. Pearson correlations between the three iWUE series were calculated per year and site.

For annual values of iWUE, we compared the means of each iWUE series for the growing periods of earlywood, latewood, and the whole tree ring. To examine the significance of interannual iWUE trends, we applied the Mann–Kendall trend test with R package ‘Kendall’ (McLeod, 2011). All statistical analyses were made in R v.4.0.0 (R Core Team, 2020).

## Results

### Comparison of intraseasonal patterns of iWUE

**Fig. 2:**
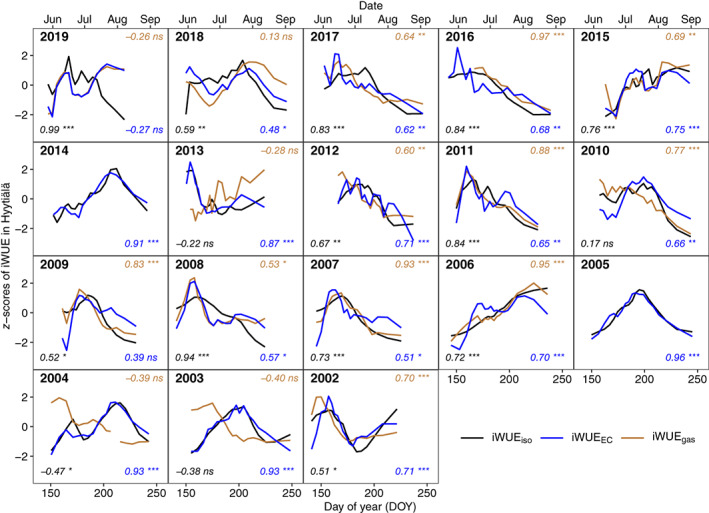
*Z*‐scores of intrinsic water‐use efficiency (iWUE) of Scots pine from 2002 to 2019 in Hyytiälä. Intrinsic water‐use efficiency was derived from gas exchange (iWUE_gas_ ), tree‐ring δ^13^C (iWUE_iso_ ), and eddy covariance (iWUE_EC_ ) data. For each year, the Pearson correlation coefficient and significance level for iWUE_gas_ and iWUE_iso_ (upper right), iWUE_EC_ and iWUE_iso_ (lower right), and iWUE_gas_ and iWUE_EC_ (lower left) are given: *, *P* < 0.05; **, *P* < 0.01; ***, *P* < 0.001; ns, not significant. Middle day of year (DOY) of the formation period representing each iWUE_iso_ data point is presented.

In Hyytiälä, iWUE_iso_ aligned in the intraseasonal trends with iWUE_EC_ and/or iWUE_gas_, except for year 2019 (Fig. 2), the year that had the smallest variations in *T* between June, July, and August (15.7°C, 15.9°C, and 15.2°C, respectively) among the studied years and had a small variability in tree‐ring δ^13^C (−27.0 ± 0.2‰). iWUE_iso_ aligned clearly better with iWUE_gas_ than with iWUE_EC_ in years 2006, 2007, 2009, 2010, 2011, and 2016, which were marked by significantly higher VPD in June compared with other years (0.74 kPa vs 0.56 kPa, *P* = 0.002). In comparison, iWUE_iso_ aligned better with iWUE_EC_ than with iWUE_gas_ in years 2003, 2004, 2013, and 2018. These years had higher soil temperature in May than the other years (6.9°C vs 5.8°C), although the difference was not significant (*P* = 0.08).

**Fig. 3:**
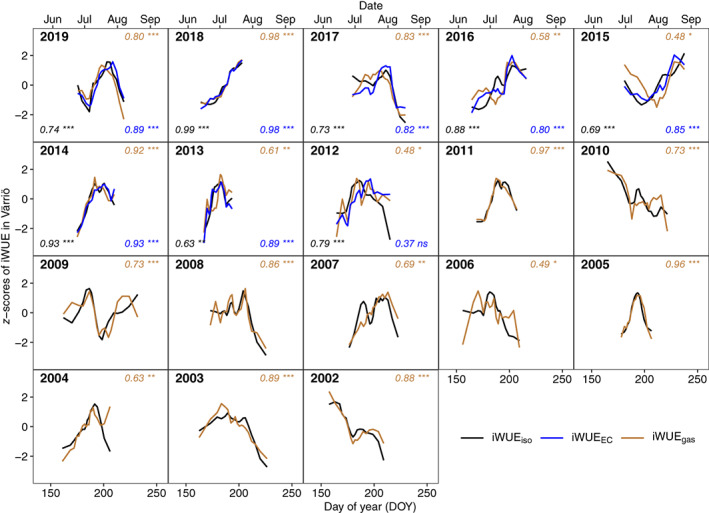
*Z*‐scores of intrinsic water‐use efficiency (iWUE) of Scots pine from 2002 to 2019 in Värriö. Intrinsic water‐use efficiency was derived from gas exchange (iWUE_gas_ ), tree‐ring δ^13^C (iWUE_iso_ ), and eddy covariance (iWUE_EC_ ) data. For each year, the Pearson correlation coefficient and significance level for iWUE_gas_ and iWUE_iso_ (upper right), iWUE_EC_ and iWUE_iso_ (lower right), and iWUE_gas_ and iWUE_EC_ (lower left) are given: *, *P* < 0.05; **, *P* < 0.01; ***, *P* < 0.001; ns, not significant. Middle day of year (DOY) of the formation period representing each iWUE_iso_ data point is presented.

In Värriö, iWUE_iso_, iWUE_EC_, and iWUE_gas_ aligned with each other in the intraseasonal trends except for year 2012 (Fig. 3). In this year, albeit showing a similar low‐frequency trend, iWUE_EC_ and iWUE_iso_ were not significantly correlated. In year 2012, the site experienced a dry period, with lowest precipitation amount in August among the studied years.

For both sites, the intraseasonal trends of iWUE_iso_ were clearly more dampened than that of iWUE_EC_ and iWUE_gas_, when absolute values instead of *z*‐scores were compared. The amplitudes of intraseasonal variations in iWUE_iso_, iWUE_gas_, and iWUE_EC_ were 13 ± 6, 24 ± 8, and 29 ± 9 ppm, respectively, in Hyytiälä, and 8 ± 3, 19 ± 6, and 30 ± 14 ppm, respectively, in Värriö. This dampened intraseasonal variation in iWUE_iso_ corresponded to a lower amplitude of intraseasonal variability in tree‐ring δ^13^C compared with leaf sucrose δ^13^C (1.3‰ vs 4.3‰ in Hyytiälä; 1.3‰ vs 4.2‰ in Värriö; Fig. S6).

### Comparison of interannual patterns of iWUE

**Fig. 4:**
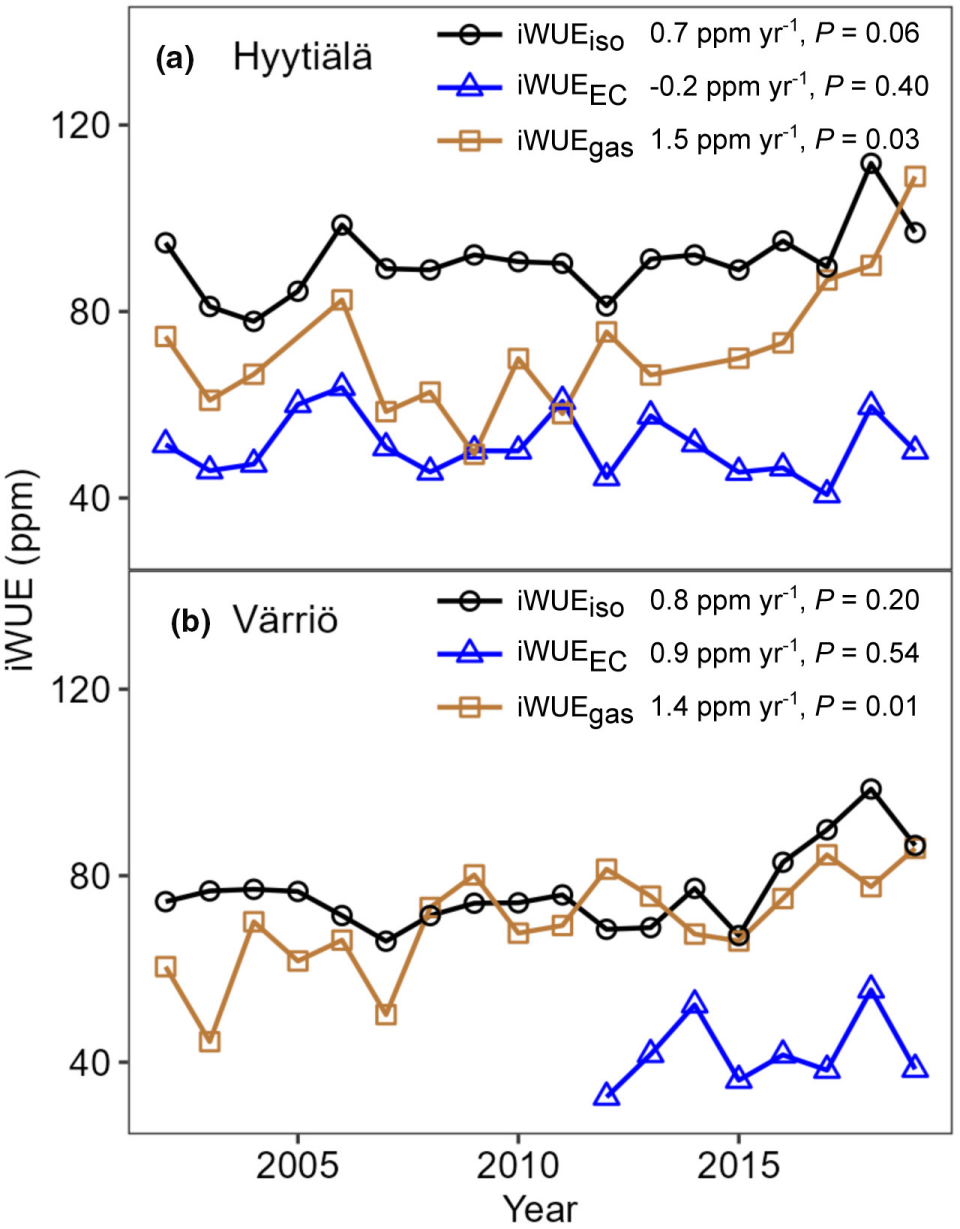
Interannual intrinsic water‐use efficiency (iWUE) of Scots pine derived from gas exchange (iWUE_gas_ ), eddy covariance (iWUE_EC_ ), and tree‐ring δ^13^C (iWUE_iso_ ) in (a) Hyytiälä and (b) Värriö from 2002 to 2019. Yearly increase rates of iWUE and *P* values of Mann–Kendall trend test are given. Pearson correlations between iWUE_iso_ and iWUE_EC_ , between iWUE_gas_ and iWUE_EC_ , and between iWUE_iso_ and iWUE_gas_ were 0.46 (*P* = 0.05), 0.48 (*P* = 0.06) and 0.05 (*P* = 0.87), respectively, in Hyytiälä; and 0.55 (*P* = 0.16), 0.42 (*P* = 0.08), and −0.31 (*P* = 0.46), respectively, in Värriö.

For each three iWUE estimates, the average absolute value of iWUE did not significantly differ between the growing periods of earlywood, latewood, and the whole ring (*P* > 0.05, Fig. S7). Hence, for examining interannual iWUE variability, we used the average iWUE values for the whole growing season. Annual iWUE_gas_ presented a statistically significant increasing trend in both Värriö (1.4 ppm yr^−1^, *P* = 0.01) and Hyytiälä (1.5 ppm yr^−1^, *P* = 0.03; Fig. 4). Annual iWUE_iso_ and iWUE_EC_ did not significantly increase during the studied period in Värriö (0.8 ppm yr^−1^, *P* = 0.20 and 0.9 ppm yr^−1^, *P* = 0.54, respectively) or Hyytiälä (0.7 ppm yr^−1^, *P* = 0.06 and −0.2 ppm yr^−1^, *P* = 0.40, respectively; Fig. 4). Annual iWUE_gas_, iWUE_iso_, and iWUE_EC_ were not significantly correlated with each other for either site (Fig. 4). Among all tested environmental variables, which were *c*_a_, RH, *T*, VPD and soil moisture, annual iWUE_gas_ correlated best with *c*_a_ at both sites (Table 1), whereas annual iWUE_iso_ and iWUE_EC_ correlated best with VPD (Table 1).

**Table 1:** Pearson correlations between environmental variables and annual intrinsic water‐use efficiency (iWUE) of Scots pine.

Site	Variables	*c*_a_	RH	SM	*T*	VPD
Hyytiälä	iWUE_gas_	0.59*	−0.19 ns	0.11 ns	0.19 ns	0.25 ns
Hyytiälä	iWUE_iso_	0.53*	−0.62**	−0.50*	0.66**	0.75***
Hyytiälä	iWUE_EC_	−0.10 ns	−0.76***	−0.54*	0.71***	0.84***
Värriö	iWUE_gas_	0.69**	0 ns	−0.04 ns	−0.25 ns	0 ns
Värriö	iWUE_iso_	0.55*	−0.50*	−0.52 ns	0.33 ns	0.58*
Värriö	iWUE_EC_	0.45 ns	−0.88**	−0.76*	0.86**	0.95***

Intrinsic water‐use efficiency was derived from gas exchange (iWUE_gas_), tree‐ring δ^13^C (iWUE_iso_), and eddy covariance (iWUE_EC_) data. *, *P* < 0.05; **, *P* < 0.01; ***, *P* < 0.001; ns, not significant. The best correlation for each category is in bold. *c*_a_, ambient CO_2_ concentration; RH, relative humidity; SM, soil moisture; *T*, temperature; VPD, vapor pressure deficit.

### Impact of *f*_post_, *g*_m_, and *f* on iWUE_iso_

To reconcile differences between iWUE_iso_, iWUE_gas_, and iWUE_gas_, we evaluated the impact of *f*_post_, *g*_m_, and *f* on iWUE_iso_.

**Fig. 5:**
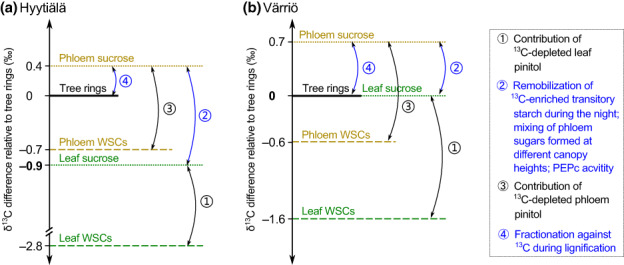
δ^13^C difference between resin‐extracted tree rings and sugar pools of Scots pine in (a) Hyytiälä and (b) Värriö, together with possible underlying mechanisms. PEPc, phospho*enol*pyruvate carboxylase; WSCs, water‐soluble carbohydrates.

**Fig. 6:**
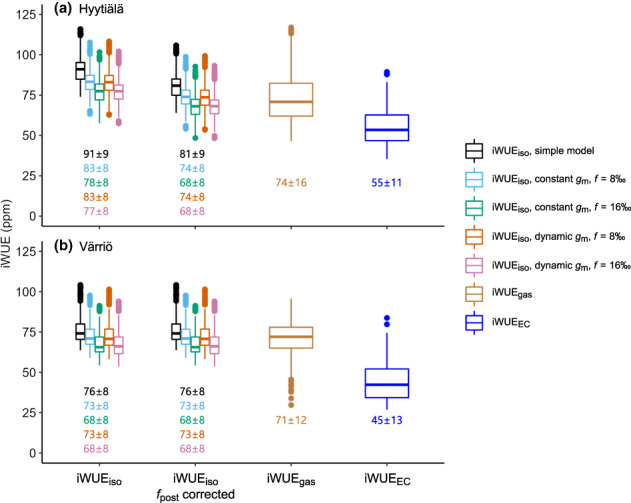
Boxplot showing the intrinsic water‐use efficiency (iWUE) of Scots pine in (a) Hyytiälä and (b) Värriö. iWUE_iso_ , iWUE_gas_, and iWUE_EC_ were derived from tree‐ring δ^13^C, gas exchange, and eddy covariance data, respectively. iWUE_iso_ was estimated from different assumptions of mesophyll conductance (*g*_m_) and photorespiration fractionation factor (*f*) and corrected by postphotosynthetic δ^13^C alteration (*f*_post_). Horizontal line represents the median, box represents the interquartile range, the tails extend to 1.5 times of the interquartile range, and dots represent outliers that are outside 1.5 times of the interquartile range. Mean ± SD value (ppm) for each category is given at the bottom.

*f*_post_ differed between the two sites, thus posing site‐specific impacts on iWUE_iso_. Among all analyzed carbon pools, leaf WSCs had the lowest δ^13^C values, whereas phloem sucrose had the highest δ^13^C values (Fig. 5). Water‐soluble carbohydrates were significantly ^13^C‐depleted in comparison with sucrose in both leaves (*P* < 0.001 for both sites) and phloem (*P* < 0.001 for both sites) (Fig. 5), due to the contribution of pinitol/*myo*‐inositol (33 ± 6% in leaves, and 18 ± 4% in phloem) with low δ^13^C values (−31.4 ± 0.4‰ in leaves, −30.6 ± 0.6‰ in phloem). In Hyytiälä, tree‐ring δ^13^C was 0.9‰ (*P* < 0.001), 2.8‰ (*P* < 0.001), and 0.7‰ (*P* = 0.03) higher than δ^13^C of leaf sucrose, leaf WSCs, and phloem WSCs, respectively, but 0.4‰ (*P* = 0.24) lower than δ^13^C of phloem sucrose (Fig. 5a). In Värriö, the δ^13^C differences from tree rings to leaf sucrose, leaf WSCs, phloem WSCs, and phloem sucrose were 0‰ (*P* = 0.99), 1.6‰ (*P* < 0.001), 0.5‰ (*P* = 0.10), and − 0.7‰ (*P* = 0.08), respectively (Fig. 5b). Common seasonal courses existed in δ^13^C variability of leaf sucrose and tree rings for both sites (Fig. S6). In Hyytiälä, both leaf sucrose δ^13^C and tree‐ring δ^13^C presented an inverse ‘V’ shape variation from June to September (Fig. S6a). In Värriö, δ^13^C of tree ring followed the general increasing trend in leaf sucrose for the whole ring formation period (Fig. S6b). Taken together, *f*_post_ from leaf sucrose to tree rings was 0.9‰ in Hyytiälä but 0.0‰ in Värriö (Fig. 5). By subtracting the 0.9‰ offset from tree‐ring δ^13^C for Hyytiälä, iWUE_iso_ decreased by 11% (Fig. 6a).

The explicit consideration of *g*_m_ and *f* in the calculation of iWUE_iso_ did not change the intraseasonal or interannual patterns of iWUE_iso_ (Fig. S8), but it lowered the absolute values of iWUE_iso_ for both sites (*P* < 0.001, Fig. 6). Constant *g*_m_ (*r* = 0.96, *P* < 0.001) and dynamic *g*_m_ (*r* = 0.97, *P* < 0.001) assumptions both produced iWUE_iso_ that linearly correlated with iWUE_iso_ from the simplified model. Similarly, iWUE_iso_ from the assumptions *f* = 8‰ (*r* = 0.93, *P* < 0.001) and *f* = 16‰ (*r* = 0.92, *P* < 0.001) both linearly correlated with iWUE_iso_ from the simplified model.

iWUE_gas_, iWUE_iso_, and iWUE_EC_ differed significantly in their absolute values for both sites (*P* < 0.05), in the following order: iWUE_iso_ > iWUE_gas_ > iWUE_EC_ (Fig. 6). In Hyytiälä, iWUE_gas_ and iWUE_EC_ were on average 19% and 39%, respectively, lower than iWUE_iso_, whereas in Värriö, the differences were 7% and 41%. If applying constant *g*_m_ and *f* = 8‰ and correcting iWUE_iso_ by *f*_post_, the differences from iWUE_iso_ to iWUE_gas_ and iWUE_EC_ decreased to 1% and 25%, respectively, in Hyytiälä; and to 3% and 39%, respectively, in Värriö (Fig. 6).

## Discussion

### Validity of tree‐ring δ^13^C for intraseasonal iWUE estimates

We observed a general agreement in the intraseasonal trends of iWUE_iso_, iWUE_gas_, and iWUE_EC_ (Figs 2, 3), which clearly supports the validity of using tree‐ring δ^13^C for estimating iWUE at high‐resolution intraseasonal scale. This is partly in line with Michelot *et al*. (2011), where the latewood section of deciduous *Quercus petraea* recorded well‐seasonal variations in iWUE. Nevertheless, our results indicate that earlywood of boreal conifers can also be a good recorder for iWUE_iso_. This is in line with Kress *et al*. (2010), although earlywood has been removed by default in some earlier annual iWUE_iso_ studies (Waterhouse *et al*., 2004) in case of possible use of reserves for earlywood growth (McCarroll & Loader, 2004). Our conclusion on the suitability of both earlywood and latewood sections for iWUE_iso_ studies is based on the following observations: variations of iWUE_iso_ during the earlywood growing period aligned with that of iWUE_EC_ and/or iWUE_gas_ (Figs 2, 3); iWUE_iso_ values averaged for the growing periods of earlywood, latewood, and the whole ring were not significantly different (Fig. S7); we did not observe a previous‐year reserve δ^13^C signal in earlywood (Fig. S9).

### Factors affecting iWUE_iso_ estimates

The impact of *f*_post_, that is, overall apparent isotope fractionation between tree rings and new assimilates, on iWUE_iso_ has been addressed in earlier studies with a correction factor that has been obtained by measuring δ^13^C difference between tree rings and leaf water‐soluble organic matter (Frank *et al*., 2015) or total organic matter (Belmecheri & Lavergne, 2020). Considering that leaf bulk materials have varying and significant δ^13^C offsets from new assimilates and that sucrose accurately records assimilate δ^13^C (Tang *et al*., in press), our comparison between tree rings and leaf sucrose presents a more precise quantification of *f*_post_. From this comparison, we defined *f*_post_ of 0.9‰ for Hyytiälä but 0.0‰ for Värriö (Fig. 5), which can reduce iWUE_iso_ by 11% in Hyytiälä (Fig. 6).

In our study, the site‐specific *f*_post_ values were not associated with site‐specificity in the use of previous‐year reserves for tree‐ring growth (Fig. S9) but may be related to site‐to‐site differences in postphotosynthetic metabolisms. First, *f*_post_ can be partly ascribed to the remobilization of ^13^C‐enriched transitory starch during the night (Gessler & Ferrio, 2022), which causes an overall ^13^C‐enrichment in phloem sucrose relative to leaf sucrose (Fig. 5). The proportion of starch‐derived ^13^C‐enriched sucrose in breast‐height phloem depends on the phloem transport velocity and tree height (Gessler & Ferrio, 2022), which differed between Hyytiälä and Värriö (Table S1). Second, *f*_post_ can be affected by the mixing of assimilates formed at different canopy heights, which may have a δ^13^C gradient of up to 8‰ (Bögelein *et al*., 2019). Apparently, the vertical mixing of assimilates should have less impact on *f*_post_ for Värriö compared with Hyytiälä, on account of a sparser canopy density and therewith lower intracanopy light gradients and assimilate δ^13^C gradients in Värriö (Fig. S1). Third, *f*_post_ is partly determined by the activity of phospho*enol*pyruvatecarboxylase (PEPc), which favors ^13^C when catalyzing CO_2_ refixation in stems (Farquhar, 1983) and thus enriches the organic matter. Since PEPc activity varies with temperature (Chinthapalli, 2003), which differed remarkably between our study sites (Table S1), *f*_post_ may have differed between the two sites. Fourth, *f*_post_ may be affected by the ratio between ^13^C‐enriched cellulose and ^13^C‐depleted lignin (Loader *et al*., 2003), which may vary between the two sites with different environmental conditions (Kilpelainen *et al*., 2005).

While the explicit consideration of *g*_m_ and *f* generally reduced iWUE_iso_ values by up to 14% (Fig. 6), it did not change the intraseasonal (Fig. S8) or interannual trends of iWUE_iso_. This is in contrast to earlier studies that have reported the potential of *g*_m_ and *f* for modulating isotopic discrimination in long term (Keeling *et al*., 2017; Lavergne *et al*., 2022). Our results thereby indicate that the simplified model for iWUE_iso_ is able to capture the intraseasonal trends of iWUE_iso_ for our study species, albeit the accuracy of absolute values of iWUE_iso_ may benefit from a better understanding of *g*_m_ dynamics in future studies. However, our results are in contrast to the report of Gimeno *et al*. (2021) for *Eucalyptus*, where a better fit between iWUE_gas_ and iWUE_iso_ was obtained after incorporating *g*_m_ into the calculations of iWUE_iso_. This contradiction probably comes from the use of a constant *g*_m_ value or temperature‐dependent *g*_m_ in this study, considering that *g*_m_ can vary with photosynthetic rate and water stress (Schiestl‐Aalto *et al*., 2021).

### Environmental and physiological controls on iWUE

Differences in intraseasonal patterns of iWUE_iso_, iWUE_EC_, and iWUE_gas_ existed in some cases, likely associated with environmental control. For instance, the intraseasonal pattern of iWUE_iso_ did not align with that of iWUE_gas_ and iWUE_EC_ for year 2019 in Hyytiälä (Fig. 2), possibly due to a dampened low‐frequency trend in tree‐ring δ^13^C data governed by low variability in *T* this year. It demonstrates that a certain degree of variability in environment conditions and tree‐ring δ^13^C data within a growing season is crucial for reliable estimation of intraseasonal iWUE_iso_. Furthermore, the intraseasonal pattern of iWUE_EC_ is impacted by water stress. For example, for years with higher VPD in Hyytiälä (Fig. 2) and for the dry year 2012 in Värriö (Fig. 3), iWUE_EC_ aligned less well with iWUE_iso_ than iWUE_gas_ did. This is probably because iWUE_EC_ integrates the species‐specific physiological response to water stress (Yi *et al*., 2019) across various plant species over the stand (Table S1). Moreover, high spring temperatures tend to cause high uncertainties in gas exchange measurements, as the measuring shoot may recover faster than the whole canopy, resulting in divergent variability in iWUE_gas_ for years 2003, 2004, 2013, and 2018 in Hyytiälä (Fig. 2).

Environmental drivers for annual iWUE were diverse across leaf (iWUE_gas_), tree (iWUE_iso_), and ecosystem (iWUE_EC_) scales (Table 1). At leaf level, rising *c*_a_ tightly regulates iWUE_gas_ (Table 1) by enhancing assimilation rate (Streit *et al*., 2014) and reducing *g*_s_ (Brodribb *et al*., 2009). However, the *c*_a_ effect on whole‐tree level iWUE_iso_ was reduced (Table 1), because the prevalent photosynthesis at lower canopy is limited by RuBP‐regeneration and less sensitive to rising *c*_a_ compared with Rubisco‐limited photosynthesis at top canopy (Yang *et al*., 2020). At ecosystem level, the *c*_a_ effect was further dampened (iWUE_EC_ in Table 1) due to species‐specific responses of iWUE to *c*_a_ (Marchand *et al*., 2020) and changes in leaf area and soil water savings (Lavergne *et al*., 2019). Instead of *c*_a_, VPD dominated changes in annual iWUE_iso_ and iWUE_EC_ (Table 1), as also identified in Kannenberg *et al*. (2021) and Zhang *et al*. (2019), respectively. This is probably because carbon uptake decreases less than *g*_s_ with increasing VPD (Zhang *et al*., 2019). We also note that environmental control on iWUE varies between interannual and intraseasonal scales and from year to year, possibly related to changes in leaf area (Launiainen *et al*., 2016) and soil moisture (Beer *et al*., 2009).

### The strengths and weaknesses of the three iWUE estimation methods

Each of the three iWUE estimation approaches has its own strengths and weaknesses (Table 2). Eddy covariance measurements have the merits in the manner that global networks, such as FLUXNET (Baldocchi *et al*., 2001), provide EC data at varying temporal coverage up to decades (Medlyn *et al*., 2017). However, as an ecosystem‐level integrated signal, EC data are not able to discern species‐specific iWUE responses (Yi *et al*., 2019). Meanwhile, iWUE_EC_ estimates have multiple sources of uncertainties (Knauer *et al*., 2018), including within‐canopy gradients, nontranspiratory water fluxes, energy balance nonclosure, issues in NEE partitioning, aerodynamic conductance, and meteorological differences between measurement height and canopy surface. Some of these uncertainties are site‐specific and may vary with time. For example, within‐canopy gradient, which may result in lower iWUE_EC_ with a higher contribution of fluxes from the understory (Domingues *et al*., 2007; Sellin *et al*., 2010), has a site‐specific impact. iWUE_EC_ calculated from subcanopy flux data was 30.5% lower than the gradient‐integrated iWUE_EC_ in Hyytiälä, whereas the difference was negligible in Värriö (6%). This site‐specific trait rises from an open stand structure in Värriö (Fig. S1), which results in lower intracanopy light gradients and a higher coupling of air exchange to the atmosphere relative to a closed canopy structure in Hyytiälä (Wieser *et al*., 2018). Moreover, iWUE_EC_ is underestimated due to the contribution of nontranspiratory water fluxes (Eqn 2), mainly soil evaporation in this study as canopy evaporation should be minimal after excluding the time periods following precipitation. This underestimation is in the order of 15%, assuming soil evaporation accounted for half of forest floor ET, which contributed to *c*. 30% of total ecosystem ET at our sites (estimated from subcanopy EC data and Launiainen *et al*., 2005). However, this proportion would change with the increase in leaf area index in Hyytiälä (Table S1) but stay almost constant over the years with roughly unchanged leaf area index in Värriö.

**Table 2:** Method comparison between intrinsic water‐use efficiency (iWUE) derived from gas exchange (iWUE_gas_), tree‐ring δ^13^C (iWUE_iso_) and eddy covariance data (iWUE_EC_).

	iWUE_gas_	iWUE_iso_	iWUE_EC_
Factors affecting iWUE estimates	(1) Possible chamber artifacts, for example, occasional mechanical flaws and inconsistence in measurement systems over years (2) Uncertainties arising from limited sampling coverage	(1) Uncertainties arising from the photosynthetic discrimination model due to, for example, limited knowledge of mesophyll conductance (2) δ^13^C alteration from leaf assimilates to tree rings, due to vertical mixing of assimilates, postphotosynthetic isotopic fractionation, and possible use of reserves	(1) Energy balance nonclosure (2) Uncertainties in net ecosystem CO_2_ exchange (NEE) partitioning (3) Within‐canopy gradient impacted by stand structure (4) Contribution of nontranspiratory water fluxes (5) Other sources of uncertainties, for example, aerodynamic conductance
Strength of the method	(1) High temporal resolution data, for example, at daily or diurnal scale (2) Information on species‐specific dynamics	(1) Signal is archived in tree materials and can be retrieved for sites (periods) where (when) no instrumental data are available years after tree‐ring formation (2) Suitable for long‐term iWUE reconstructions (3) No need for on‐site δ^13^C measurements (4) Information on species‐specific dynamics	(1) High temporal resolution data (2) Continuous and long‐term EC records are available for many sites globally
Weakness of the method	(1) Labor‐intensive and requires accessing leaves of tall trees (2) Restricted in their spatial and temporal coverage (3) Low consistency in chamber systems in long term (4) Chamber systems are prone to mechanical flaws	(1) May be influenced by other sources of isotopic discrimination	(1) Can not resolve species‐specific leaf‐ or tree‐scale dynamics (2) Subject to noise and errors in, for instance, unclosed energy balance problem and NEE partition

Leaf gas exchange measurements have the advantages of tracing instantaneous changes in iWUE, but this method is labor‐intensive and requires accessing leaves of tall trees (Yi *et al*., 2019). Meanwhile, even though a global compilation of gas exchange measurements is available (Lin *et al*., 2015), there is currently a lack of long‐term continuous datasets. Also, iWUE_gas_ estimates are subject to uncertainties regarding chamber artifacts, limited sampling coverage, and low consistency in measurement systems in long term. Occasional mechanical flaws, for example leaks, and possible damages to the measuring shoots may affect the observed seasonal cycle. Limited sampling coverage on one or several measuring shoots on the top canopy may induce uncertainties, for example, during warm springs. Changes in measurement systems over years may bias the interannual trend of iWUE_gas_. Nevertheless, not including boundary layer conductance in calculating iWUE_gas_ overall has a limited impact on absolute values (Seibt *et al*., 2008) and intraseasonal patterns of iWUE_gas_ (Figs 2, 3).

Tree‐ring δ^13^C can be retrieved even decades or centuries after tree‐ring formation without laborious work on site (Cernusak, 2020). Hence, a major advantage of tree‐ring δ^13^C records is their potential for reconstructing long‐term iWUE_iso_. Accuracy of iWUE_iso_ estimates can be further improved by a better understanding of *f*_post_, *g*_m_, and *f*. More importantly, our study shows that iWUE can be obtained from tree‐ring δ^13^C at intraseasonal scale with reasonable effort using LA‐IRMS, extending the application of this iWUE estimation method from annual resolution to intraseasonal resolution. This finding is inspiring in the way that it provides a valuable method for intraseasonal iWUE estimates, especially for sites and periods where and when no gas exchange or EC data are available.

### Conclusions

This work presented the first comparison between intraseasonal and interannual iWUE signal derived from leaf gas exchange, tree‐ring δ^13^C, and EC data, resting on a unique set of 18‐yr‐long records in two boreal forest sites. The alignment in intraseasonal iWUE trends across different methods demonstrated the reliability of tree‐ring δ^13^C derived intraseasonal iWUE estimates. This result is of special significance to studies, which seek to detect intraseasonal tree physiological dynamics in terms of iWUE but with no access to instrumental data. The absolute values of iWUE across different datasets can be reconciled by taking into account an overestimation in iWUE_iso_ of up to 11% due to *f*_post_, and of up to 14% due to nonexplicit consideration of mesophyll and photorespiratory effects. A significant increasing interannual trend existed in iWUE_gas_, but not in iWUE_iso_ or iWUE_EC_, for both sites, possibly resulting from a predominant control of *c*_a_ on iWUE_gas_ but VPD control on iWUE_iso_ and iWUE_EC_. We encourage more across‐method comparisons of iWUE at various temporal and spatial scales in the future. Such studies will not only deepen our understanding of how trees physiologically adapt to climate change but also provide insights into ecological models in respect of linking ecological information across scales.

## Competing interests

None declared.

## Author contributions

KTR‐G and YT planned and designed the study. YT, PS‐A, KTR‐G, ES and GY conducted fieldwork. YT prepared sugar samples for δ^13^C analysis. GY prepared tree‐ring samples for δ^13^C analysis. ES conducted bulk and LA‐IRMS δ^13^C analysis. MS conducted HPLC‐IRMS δ^13^C analysis. PK calculated eddy covariance and leaf gas exchange data. PS‐A modeled tree‐ring growth via CASSIA. YT and TJ conducted xylogenesis observations. YT conducted data analysis. YT was responsible for writing the manuscript. JB and all other authors contributed to the interpretation of data and the writing of the manuscript at various stages.

## Supporting information


**Fig. S1** Locations and photographs of the study sites in Finland.
**Fig. S2** Intraseasonal tree‐ring δ^13^C of Scots pine from 2002 to 2019 in Hyytiälä.
**Fig. S3** Intraseasonal tree‐ring δ^13^C of Scots pine from 2002 to 2019 in Värriö.
**Fig. S4** Comparison of growth curves of Scots pine from CASSIA model and from xylogenesis observations.
**Fig. S5** Relationship between event‐based δ^13^C of atmospheric CO_2_ (δ^13^C_air_) and concentration of ambient CO_2_ (*c*_a_) in Pallas.
**Fig. S6** Comparison of δ^13^C signal in leaf sugars, phloem sugars, and resin‐extracted wood of Scots pine in Hyytiälä and Värriö in 2018.
**Fig. S7** Boxplot showing the intrinsic water‐use efficiency of Scots pine averaged for the growing periods of earlywood, latewood, and whole ring.
**Fig. S8** Boxplot showing correlations between intraseasonal intrinsic water‐use efficiency of Scots pine derived from different methods under different mesophyll and photorespiratory assumptions.
**Fig. S9** Across‐border correlations in tree‐ring δ^13^C of Scots pine, which denotes the degree of use of previous‐year reserves.
**Methods S1** LA‐IRMS systems.
**Methods S2** Tracheid growth curves from xylogenesis observations and CASSIA model.
**Methods S3** Dynamic *g*_m_ assumption.
**Table S1** General description, site characteristics, and data availability for our study sites.
Please note: Wiley is not responsible for the content or functionality of any Supporting Information supplied by the authors. Any queries (other than missing material) should be directed to the *New Phytologist* Central Office.

## Data Availability

The data that support the findings of this study are openly available in Figshare at doi: 10.6084/m9.figshare.21267963.v1.
