# Supplementary material for: Estimating intraseasonal intrinsic water‐use efficiency from high‐resolution tree‐ring δ13C data in boreal Scots pine forests
Source: New Phytol. 2022 Dec 19;237(5):1606–19. doi: 10.1111/nph.18649 (PMC10108005; doi:10.1111/nph.18649)
Supplement: Supplementary file 1 — Fig. S1 Locations and photographs of the study sites in Finland. Fig. S2 Intraseasonal tree‐ring δ13C of Scots pine from 2002 to 2019 in Hyytiälä. Fig. S3 Intraseasonal tree‐ring δ13C of Scots pine from 2002 to 2019 in Värriö. Fig. S4 Comparison of growth curves of Scots pine from CASSIA model and from xylogenesis observations. Fig. S5 Relationship between event‐based δ13C of atmospheric CO2 (δ13Cair) and concentration of ambient CO2 (c a) in Pallas. Fig. S6 Comparison of δ13C signal in leaf sugars, phloem sugars, and resin‐extracted wood of Scots pine in Hyytiälä and Värriö in 2018. Fig. S7 Boxplot showing the intrinsic water‐use efficiency of Scots pine averaged for the growing periods of earlywood, latewood, and whole ring. Fig. S8 Boxplot showing correlations between intraseasonal intrinsic water‐use efficiency of Scots pine derived from different methods under different mesophyll and photorespiratory assumptions. Fig. S9 Across‐border correlations in tree‐ring δ13C of Scots pine, which denotes the degree of use of previous‐year reserves. Methods S1 LA‐IRMS systems. Methods S2 Tracheid growth curves from xylogenesis observations and CASSIA model. Methods S3 Dynamic g m assumption. Table S1 General description, site characteristics, and data availability for our study sites. Please note: Wiley is not responsible for the content or functionality of any Supporting Information supplied by the authors. Any queries (other than missing material) should be directed to the New Phytologist Central Office. [file NPH-237-1606-s001.pdf]

New Phytologist **Supporting Information**

Article title: Estimating intraseasonal intrinsic water-use efficiency from high-resolution tree-ring $\delta^{13}C$ data in boreal Scots pine forests

Authors: Yu Tang, Elina Sahlstedt, Giles Young, Pauliina Schiestl-Aalto, Matthias Saurer, Pasi Kolari, Tuula Jyske, Jaana Bäck, Katja T. Rinne-Garmston

Article acceptance date: 16 November 2022

The following Supporting Information is available for this article:

Fig. S1 Locations and photographs of the study sites in Finland.

Fig. S2 Intraseasonal tree-ring $\delta^{13}C$ of Scots pine from 2002 to 2019 in Hyytiälä.

Fig. S3 Intraseasonal tree-ring $\delta^{13}C$ of Scots pine from 2002 to 2019 in Värriö.

Fig. S4 Comparison of growth curves of Scots pine from CASSIA model and from xylogenesis observations.

Fig. S5 Relationship between event-based $\delta^{13}C$ of atmospheric CO_2 ($\delta^{13}C_{air}$) and concentration of ambient CO_2 (c_a) in Pallas.

Fig. S6 Comparison of $\delta^{13}C$ signal in leaf sugars, phloem sugars and resin-extracted wood of Scots pine in (a) Hyytiälä and (b) Värriö in 2018.

Fig. S7 Boxplot showing the intrinsic water-use efficiency (iWUE) of Scots pine averaged for the growing periods of earlywood, latewood and whole ring.

Fig. S8 Boxplot showing correlations between intraseasonal intrinsic water-use efficiency (iWUE) of Scots pine derived from different methods under different mesophyll and photorespiratory assumptions.

Fig. S9 Across-border correlations in tree-ring $\delta^{13}C$ of Scots pine, which denotes the degree of use of previous-year reserves.

Table S1 General description, site characteristics and data availability for our study sites.

Methods S1 LA-IRMS system

Methods S2 Tracheid growth curves from xylogenesis observations and CASSIA model

Methods S3 Dynamic g_m assumption

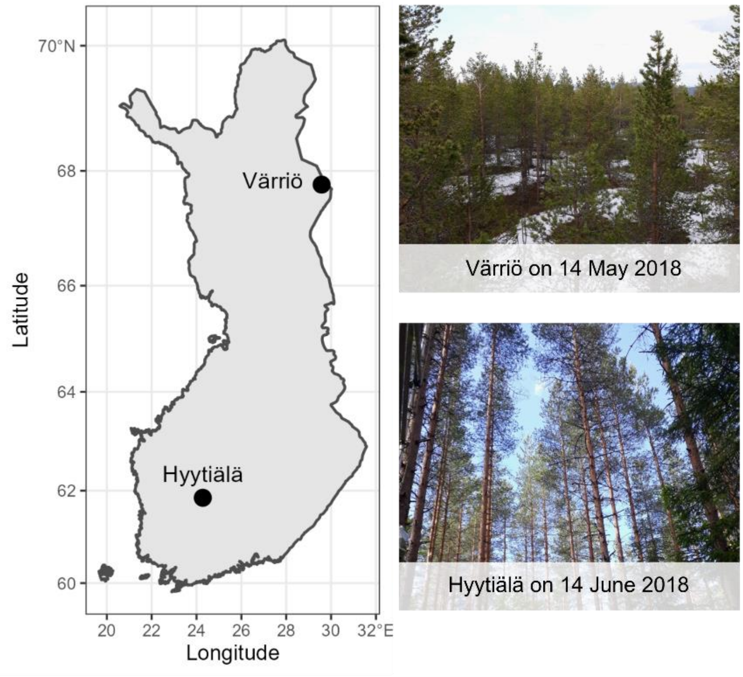

Fig. S1 Locations and photographs of the study sites in Finland.

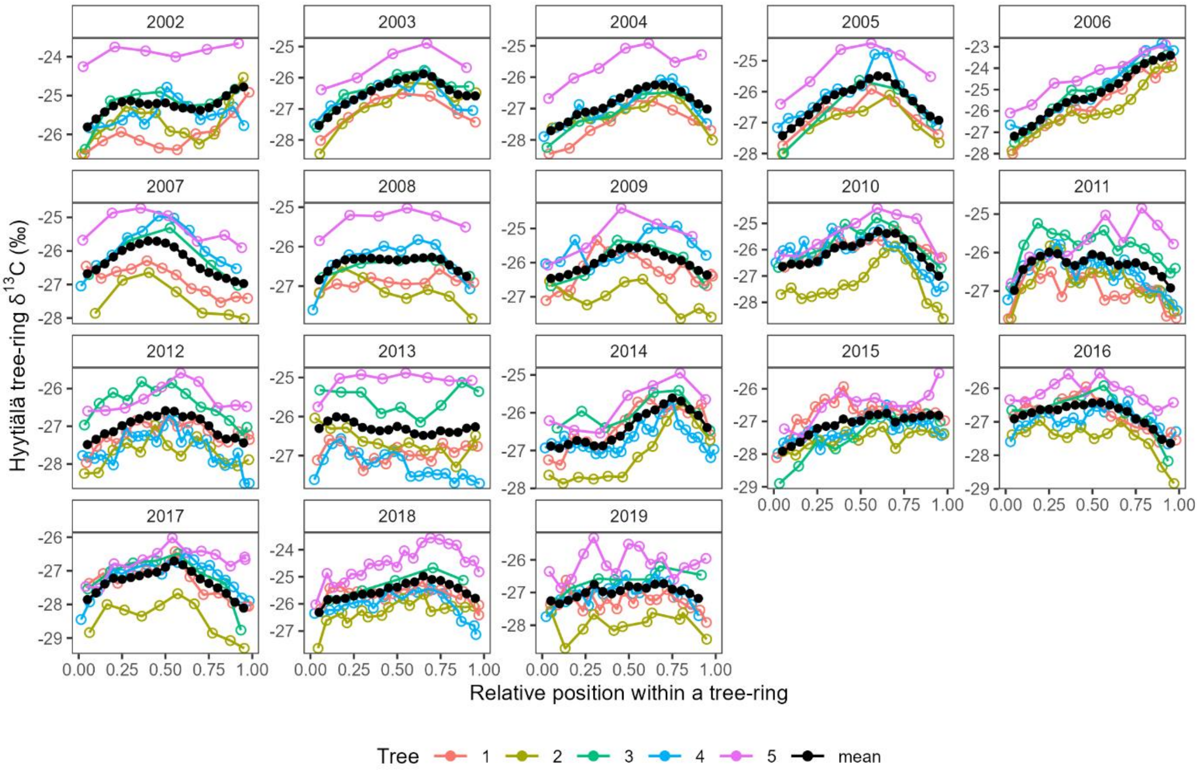

Fig. S2 Intraseasonal tree-ring $\delta^{13}C$ of Scots pine from 2002 to 2019 in Hyytiälä. Individual tree data are shown in color, and interpolated site-representative mean values are in black.

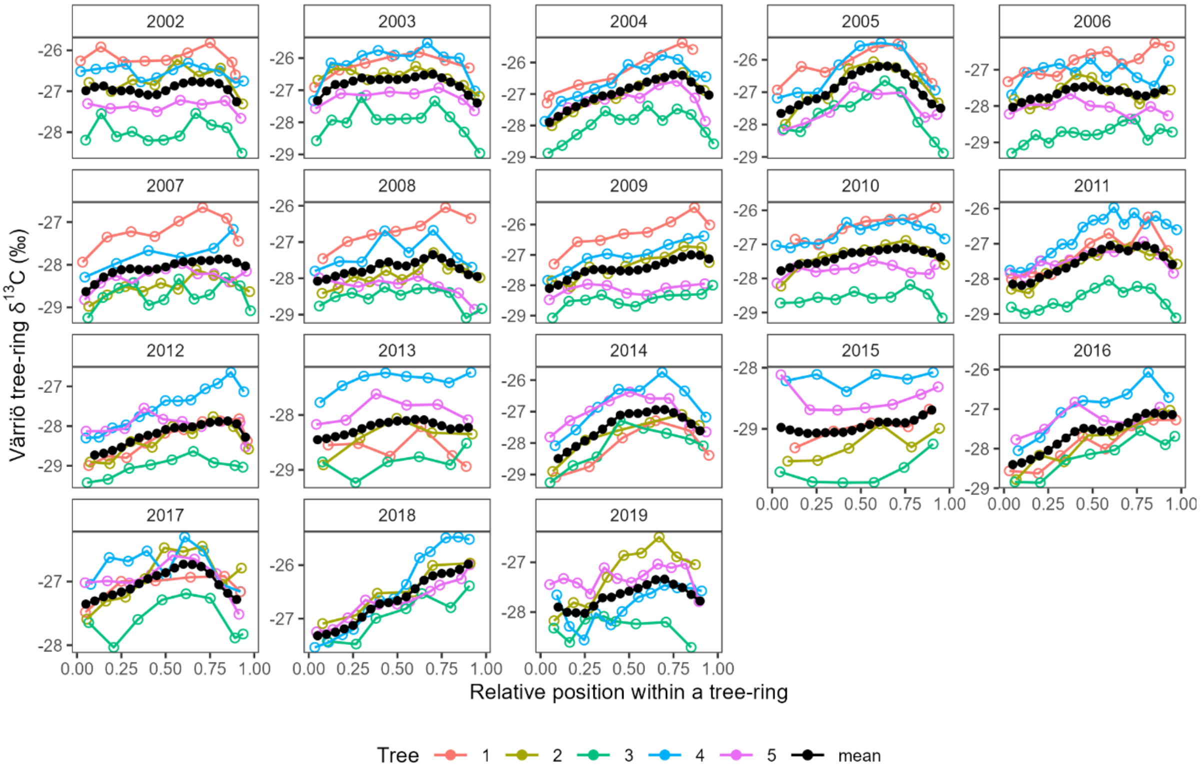

Fig. S3 Intraseasonal tree-ring δ^{13}C of Scots pine from 2002 to 2019 in Värriö. Individual trees data are shown in color, and interpolated site-representative mean values are in black.

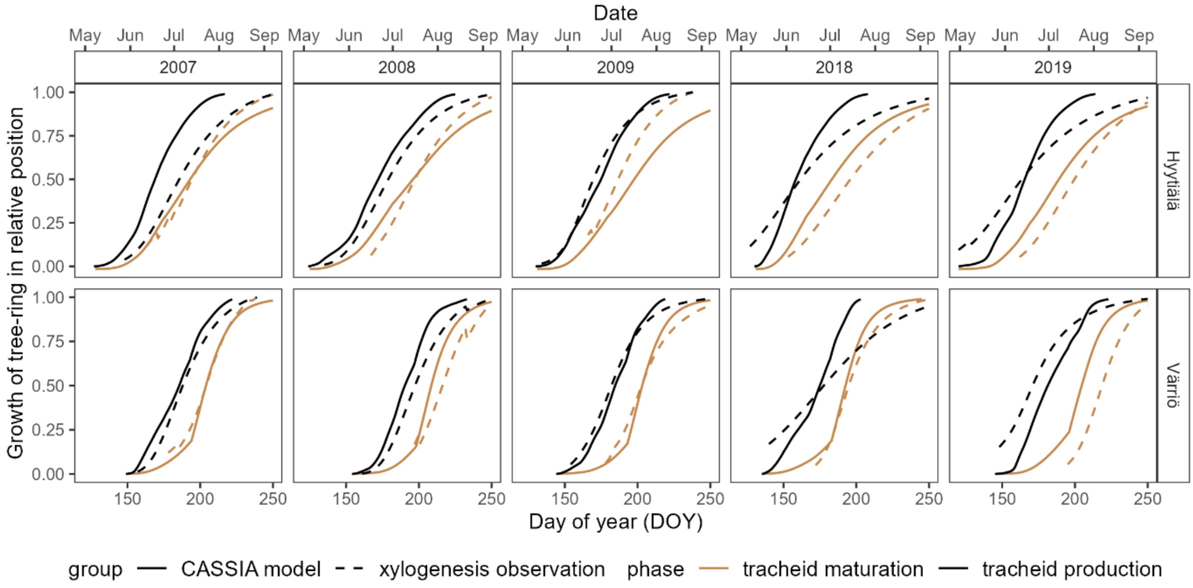

Fig. S4 Comparison of growth curves of Scots pine from CASSIA model (Schiestl-Aalto *et al.*, 2015) and from xylogenesis observations.

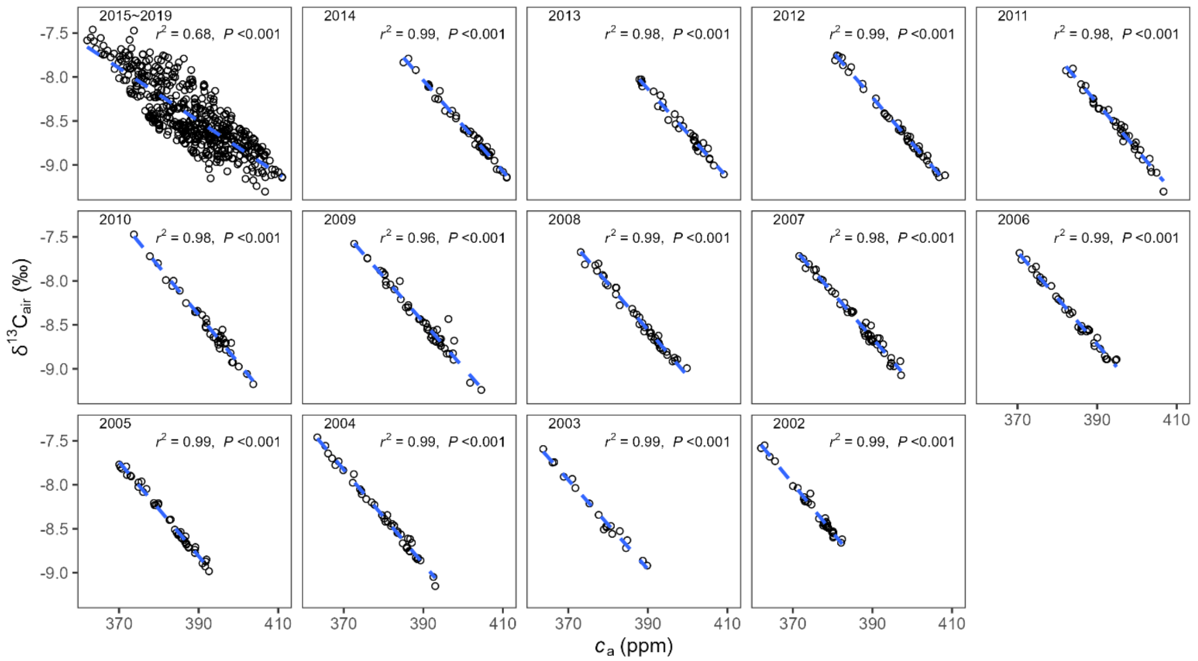

Fig. S5 Relationship between event-based $\delta^{13}C$ of atmospheric CO_2 ($\delta^{13}C_{air}$) and concentration of ambient CO_2 (c_a) in Pallas. For years from 2015 to 2019, when there were no $\delta^{13}C_{air}$ data observed, we used the relationship based on all observations from previous years. r^2 and P values of Pearson correlations between $\delta^{13}C_{air}$ and c_a are given.

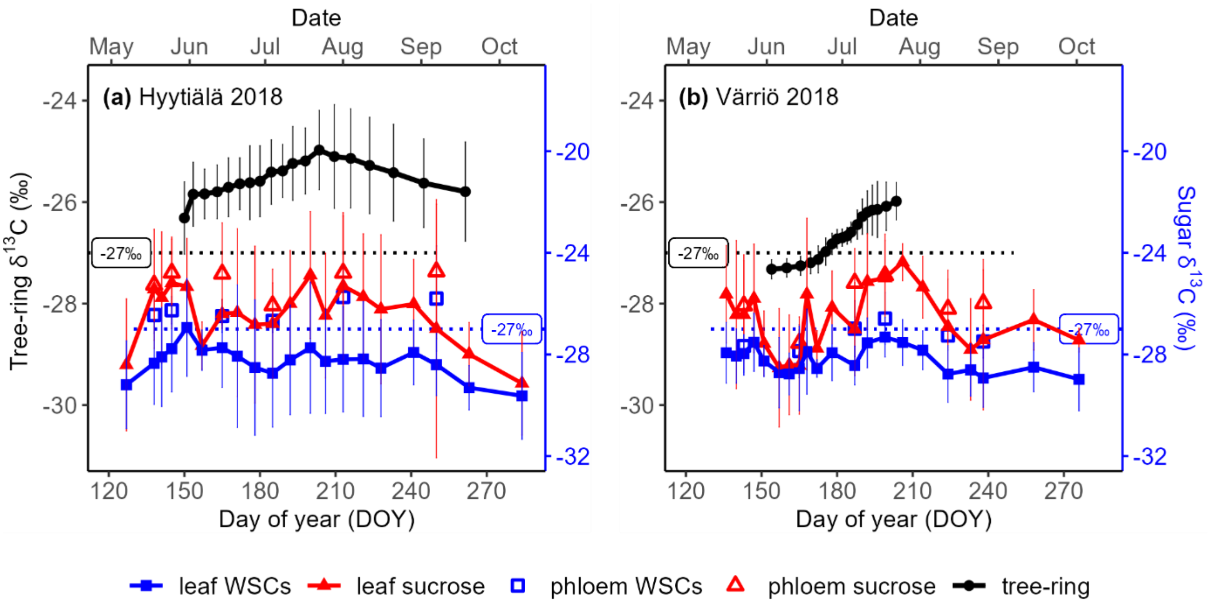

Fig. S6 Comparison of $\delta^{13}C$ signal in leaf sugars, phloem sugars and resin-extracted wood of Scots pine in (a) Hyytiälä and (b) Värriö in 2018. Middle day of year (DOY) of the formation period representing each tree-ring $\delta^{13}C$ data is represented. WSCs is water-soluble carbohydrates. Error bars represent SD from five trees.

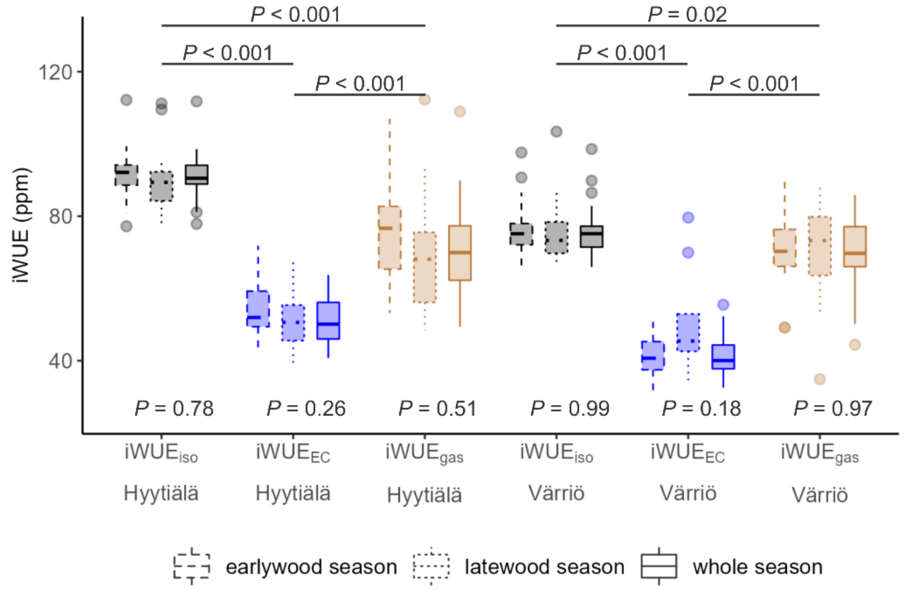

Fig. S7 Boxplot showing the intrinsic water-use efficiency (iWUE) of Scots pine averaged for the growing periods of earlywood, latewood and whole ring. iWUE was derived from gas exchange ($iWUE_{gas}$), tree-ring $\delta^{13}C$ ($iWUE_{iso}$) and eddy covariance data ($iWUE_{EC}$). Horizontal line represents the median, box represents the interquartile range, the tails extend to 1.5 times of the interquartile range, and dots represent outliers that are outside 1.5 times of the interquartile range. *P* values (ANOVA) testing the difference in iWUE averaged from different seasons are shown at the bottom; *P* values (ANOVA) testing the difference in iWUE derived from different datasets are shown above.

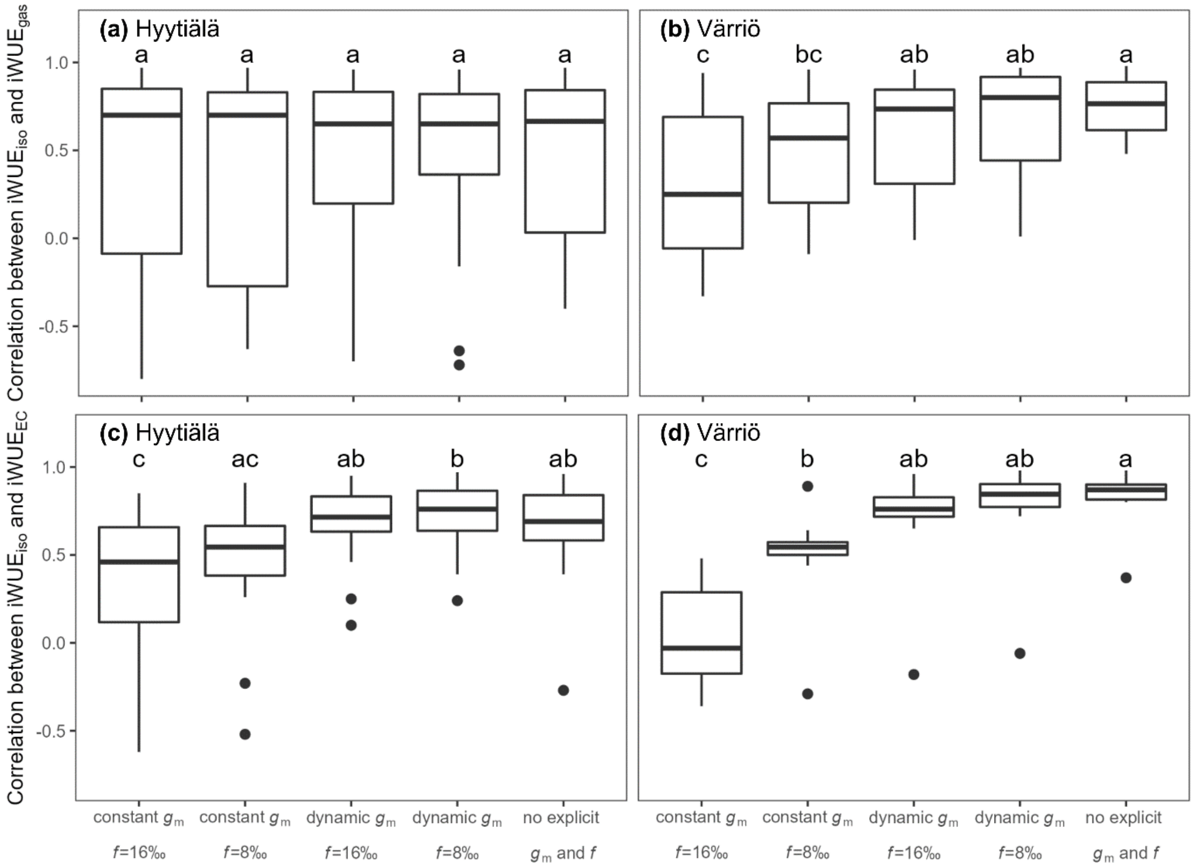

Fig. S8 Boxplot showing correlations between intraseasonal intrinsic water-use efficiency (iWUE) of Scots pine derived from different methods under different mesophyll and photorespiratory assumptions. iWUE was derived from gas exchange ($iWUE_{gas}$), tree-ring $\delta^{13}C$ ($iWUE_{iso}$) and eddy covariance data ($iWUE_{EC}$). g_m is mesophyll conductance, and f is the fractionation during photorespiration. Horizontal line represents the median, box represents the interquartile range, the tails extend to 1.5 times of the interquartile range, and dots represent outliers that are outside 1.5 times of the interquartile range. Letters indicate different correlation coefficient across different assumptions (t-test).

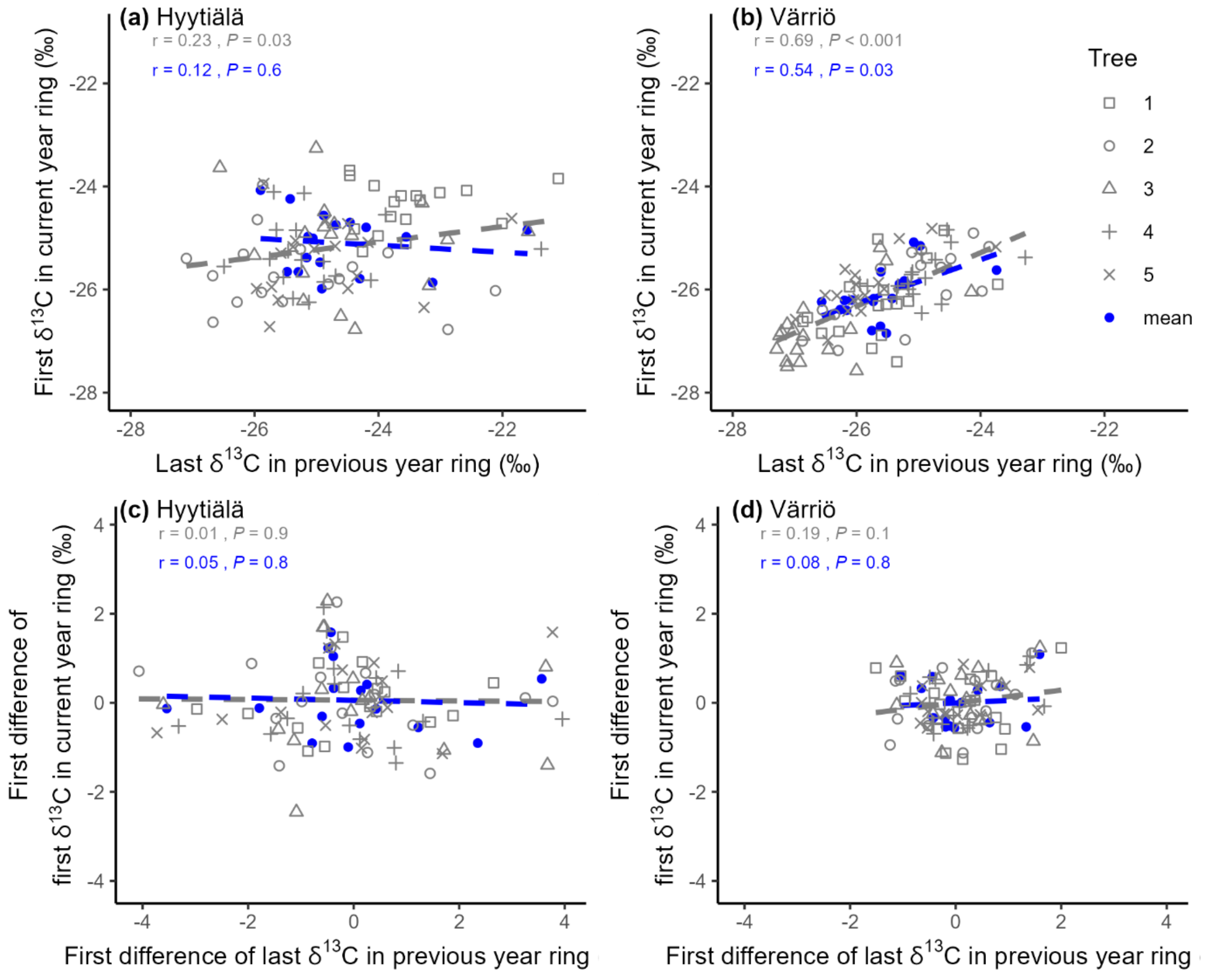

Fig. S9 Across-border correlations in tree-ring δ^{13}C of Scots pine, which denotes the degree of use of previous-year reserves (Fonti *et al.*, 2018). y axis is the first δ^{13}C observation in the current-year tree ring and x axis is the last δ^{13}C observation in previous-year tree ring. Pearson correlation coefficient and *P* value between x and y values are given for individual trees (blue) and site-representative mean values. In (a) and (b), the tree-ring δ^{13}C data were corrected by the trend in δ^{13}C of atmospheric CO_2. In (c) and (d), the low-frequency trends in tree-ring δ^{13}C data were removed by first differencing, as suggested by McCarroll *et al.* (2017). (a) and (c) are for Hyytiälä, (b) and (d) are for Värriö. As the correlations in (c) and (d) became insignificant after first differencing, we concluded that the correlations in (a) and (b) were spurious, caused by annual trends instead of use of previous-year reserves.

Table S1 General description, site characteristics and data availability for our study sites.

Site	Location	Precipitation	Temperature	Soil	Forest
Hyytiälä	61°51′N, 24°17′E, 170 m a.s.l.	711 mm per year, with 35% distributed during June to August	3.5°C annual mean, varying from –7.7°C in February to 16.0°C in July	Haplic podzol, with a mineral soil layer depth of 0.5–0.7 m and an average depth of organic layer 5.4 cm	Managed forest dominated by *Pinus sylvestris* L., mixed with Norway spruce (*Picea abies* (L.) Karst) and birches (*Betula pubescens* Ehrh. and *Betula pendula* Roth)
Värriö	67°46′N, 29°35′E, 400 m a.s.l.	601 mm per year, with 37% distributed during June to August	–0.5°C annual mean, varying from –11.4°C in January to 13.1°C in July	Haplic podzol with sand tills, with a 0–2.2 cm top layer, a 0–1.2 cm humus layer, an 1–4.5 cm eluvial horizon and an 1–6 cm illuvial horizon	Unmanaged homogenous *Pinus sylvestris* L. stand, with a variety of mosses, lichen and dwarf shrubs in the forest floor

Site	Stand density	Length of growing season	Tree height	Tree age	Half-total leaf area index (m^2 m^{-2})
Hyytiälä	1304 ha^{-1}	150 d	~20 m	~60 yr	Increased steadily from 3.2 m^2 m^{-2} in 2002 to 4.6 m^2 m^{-2} in 2018
Värriö	750 ha^{-1}	105~120 d	~10 m	~60 yr	Kept around 1.6 m^2 m^{-2} over the years

Data were from Pirinen *et al.* (2012), Köster *et al.* (2014), Kulmala *et al.* (2019), Kolari *et al.* (2022).

Methods S1 LA-IRMS system

The LA-IRMS system is comprised of a laser unit (213 nm UV laser, LSX-213 G2+, by Teledyne Photon Machines), a combustion unit, a CO_2 collection unit and an IRMS. The laser ablation system is equipped with a custom-made laser cell with an inner chamber volume of circa 6 cm^3 (TerraAnalytic). The laser is operated using separate software that allows the user to select appropriate lasing settings (laser energy, fire mode, spot size, scan speed etc.) and set up a sampling sequence for the analytical run. For the analysis of resin-extracted wood, we found the following settings to be appropriate: laser energy of 40% (8 J cm^{-2} fluence), repetition rate 20 Hz, spot size 40 µm and scan speed of 10 µm s^{-1}, run on a 300 µm track length. The particles released from the sample surface were carried by a flow of He (60 ml min^{-1}) via PEEK capillary tubing into a 6 mm OD quartz tube reactor filled with Cr_2O_3 and held at 700°C. The combustion unit releases the sample carbon as CO_2 which was collected in liquid N_2 trap (CryoPrep-2 unit, Sercon Ltd., Crewe, UK). Water vapour released from the sample during combustion process was separated from the sample flow by Nafion membrane located downstream from the combustion unit. Before releasing the sample CO_2 gas from the liquid N_2 trap, the He carrier gas flow was lowered to about 10 ml min^{-1} via 6-way Valco valve. Sample CO_2 gas was released as a pulse from the liquid N_2 trap by heating the capillary back into room temperature, after which the sample travels via gas chromatographic column to the IRMS (20-22 Stable Isotope Mass Spectrometer, Sercon Ltd.) for the measurement of its isotope composition.

Methods S2 Tracheid growth curves from xylogenesis observations and CASSIA model

Micro-cores up to 15 mm were taken from four to five mature Scots pine trees at breast height during years 2007-2009 and 2018-2019 at both sites. 20 to 27 samples were taken per year with higher sampling frequency in early growing season and lower frequency towards the end of the growing season. In laboratory, 8 μm transverse micro-core sections were prepared and analyzed to determine the number of current-year tracheids in the enlargement, wall-thickening and lignification, and mature phases (Jyske *et al.*, 2014). The growth curves for tracheid production and tracheid maturation were obtained via Gompertz fitting (Zeide, 1993) on the number of total and mature current-year tracheids, respectively, using the 'nlsLM' function of R package 'minpack.lm' (Elzhov *et al.*, 2010).

Meanwhile, the dimensional growth curve of tracheid production and the growth curve of mature tracheid number were modeled in CASSIA with input parameters validated for Hyytiälä and Värriö (Schiestl-Aalto *et al.*, 2015). Based on tracheid cell dimensional measurements in radial direction (Jyske *et al.*, 2014), we constructed a non-linear fitting curve between cell number and tree-ring dimension in CurveExpert 1.6.0 and transferred the number-based growth curves to dimensional growth curves. We evaluated the quality of CASSIA model results via comparing them with the observational results (Fig. S4).

However, there are several sources of uncertainties in tracheid growth prediction via the CASSIA model (Fig. S4), which uses air T as the main driver. First, even though temperature response function has proven to be good in most conditions (Schiestl-Aalto *et al.*, 2015), it may underestimate or overestimate growth or development rate during long lasting unusually cold or warm periods. Second, although temperature is the most important factor determining tracheid growth (Jyske *et al.*, 2014), other factors, such as photoperiod (Rossi *et al.*, 2006) or water availability (Gruber *et al.*, 2010), may also affect growth rhythm. Third, the model applies a simplistic description of different growth phases (Schiestl-Aalto *et al.*, 2015), and thus the timings of, for example, enlargement and lignification period in relation to each other, may not be fully considered.

Considering the uncertainties in tracheid growth modeling, the modeled growth curves were shifted by at most 10 d per year and site. The results which gave best intraseasonal alignment between $iWUE_{iso}$, $iWUE_{gas}$ and $iWUE_{EC}$ were reported.

Methods S3 Dynamic g_m assumption

Mesophyll conductance (g_m) dynamics are set as function of temperature (S1.1, S1.2), according to Sun *et al.* (2014).

$$g_{\mathrm{m}}(t) = g_m^0 + g_m^{25} \cdot r_{\mathrm{T}}(t) \cdot r_{\mathrm{I}} \tag{S1.1}$$

$$r_T(t) = \exp[(c - \Delta H_\alpha/(R \cdot T_l)]/\{1 + \exp\,[(\Delta S \cdot T_l - \Delta H_d)/(R \cdot T_l)]\} \tag{S1.2}$$

where g_m^{25}=0.2 mol m^{-2} s^{-1} (Schiestl-Aalto *et al.*, 2021), corrected for all-sided leaf area; c is a scaling constant, 20.0; ΔH_α is the activation energy, 49.6 × 10^3 J mol^{-1}; ΔH_d is the deactivation energy, 437.4 × 10^3 J mol^{-1}; ΔS is an entropy term, 1.4 × 10^3 J mol^{-1} K^{-1}; R is the universal gas constant, 8.314 J mol^{-1} K^{-1}; T_l is the leaf temperature in K, taken as air temperature; r_{I}=0.96 for

top canopy (Schiestl-Aalto *et al.*, 2021). g_m^0 was set to 0.037 mol m^{-2} s^{-1} for Värriö and 0.015 mol m^{-2} s^{-1} for Hyytiälä so that the mean value of dynamic g_m during the study period for each site was equal to the constant g_m used in this study, i.e., 0.127 mol m^{-2} s^{-1} (Stangl *et al.*, 2019).

References

Elzhov T, Mullen K, Spiess AN, Bolker B. **2010**. *R interface to the Levenberg-Marquardt nonlinear least-squares algorithm found in MINPACK, Plus support for bounds, v.1.2-2*. URL https://CRAN.R-project.org/package=minpack.lm

Fonti M, Vaganov E, Wirth C, Shashkin A, Astrakhantseva N, Schulze E-D. **2018**. Age-effect on intra-annual δ^{13}C-variability within Scots pine tree-rings from central Siberia. *Forests* **9**: 364.

Gruber A, Strobl S, Veit B, Oberhuber W. **2010**. Impact of drought on the temporal dynamics of wood formation in *Pinus sylvestris*. *Tree Physiology* **30**: 490–501.

Jyske T, Mäkinen H, Kalliokoski T, Nöjd P. **2014**. Intra-annual tracheid production of Norway spruce and Scots pine across a latitudinal gradient in Finland. *Agricultural and Forest Meteorology* **194**: 241–254.

Kolari P, Aalto J, Levula J, Kulmala L, Ilvesniemi H, Pumpanen J. 2022. SMEAR II Hyytiälä site characteristics. *Zenodo*. doi: 10.5281/zenodo.5909681

Köster K, Berninger F, Lindén A, Köster E, Pumpanen J. **2014**. Recovery in fungal biomass is related to decrease in soil organic matter turnover time in a boreal fire chronosequence. *Geoderma* **235–236**: 74–82.

Kulmala L, Pumpanen J, Kolari P, Dengel S, Berninger F, Köster K, Matkala L, Vanhatalo A, Vesala T, Bäck J. **2019**. Inter- and intra-annual dynamics of photosynthesis differ between forest floor vegetation and tree canopy in a subarctic Scots pine stand. *Agricultural and Forest Meteorology* **271**: 1–11.

McCarroll D, Whitney M, Young GHF, Loader NJ, Gagen MH. **2017**. A simple stable carbon isotope method for investigating changes in the use of recent versus old carbon in oak. *Tree Physiology* **37**: 1021–1027.

Pirinen P, Simola H, Aalto J, Kaukoranta J, Karlsson P, Ruuhela R. **2012**. *Climatological statistics of Finland 1981-2010.* Finnish Meteorological Institute, Reports 2012. Helsinki, Finland: Finnish Meteorological Institute.

Rossi S, Deslauriers A, Anfodillo T, Morin H, Saracino A, Motta R, Borghetti M. **2006**. Conifers in cold environments synchronize maximum growth rate of tree-ring formation with day length. *New Phytologist* **170**: 301–310.

Schiestl-Aalto P, Kulmala L, Mäkinen H, Nikinmaa E, Mäkelä A. **2015**. CASSIA – A dynamic model for predicting intra-annual sink demand and interannual growth variation in Scots pine. *New Phytologist* **206**: 647–659.

Schiestl-Aalto P, Stangl ZR, Tarvainen L, Wallin G, Marshall J, Mäkelä A. **2021**. Linking canopy-scale mesophyll conductance and phloem sugar $\delta^{13}C$ using empirical and modelling approaches. *New Phytologist* **229**: 3141–3155.

Stangl ZR, Tarvainen L, Wallin G, Ubierna N, Räntfors M, Marshall JD. **2019**. Diurnal variation in mesophyll conductance and its influence on modelled water-use efficiency in a mature boreal *Pinus sylvestris* stand. *Photosynthesis Research* **141**: 53–63.

Sun Y, Gu L, Dickinson RE, Norby RJ, Pallardy SG, Hoffman FM. **2014**. Impact of mesophyll diffusion on estimated global land CO_2 fertilization. *Proceedings of the National Academy of Sciences* **111**: 15774–15779.

Zeide B. **1993**. Analysis of growth equations. *Forest Science* **39**: 594–616.
